# PKA antagonizes CLASP-dependent microtubule stabilization to re-localize Pom1 and buffer cell size upon glucose limitation

**DOI:** 10.1038/ncomms9445

**Published:** 2015-10-07

**Authors:** Manasi Kelkar, Sophie G. Martin

**Affiliations:** 1 Department of Fundamental Microbiology, University of Lausanne, Biophore Building, CH-1015 Lausanne, Switzerland

## Abstract

Cells couple growth with division and regulate size in response to nutrient availability. In rod-shaped fission yeast, cell-size control occurs at mitotic commitment. An important regulator is the DYRK-family kinase Pom1, which forms gradients from cell poles and inhibits the mitotic activator Cdr2, itself localized at the medial cortex. Where and when Pom1 modulates Cdr2 activity is unclear as Pom1 medial cortical levels remain constant during cell elongation. Here we show that Pom1 re-localizes to cell sides upon environmental glucose limitation, where it strongly delays mitosis. This re-localization is caused by severe microtubule destabilization upon glucose starvation, with microtubules undergoing catastrophe and depositing the Pom1 gradient nucleator Tea4 at cell sides. Microtubule destabilization requires PKA/Pka1 activity, which negatively regulates the microtubule rescue factor CLASP/Cls1/Peg1, reducing CLASP’s ability to stabilize microtubules. Thus, PKA signalling tunes CLASP’s activity to promote Pom1 cell side localization and buffer cell size upon glucose starvation.

## Abstract (web summary)

In fission yeast, cell growth is co-ordinated with division by the cell tip-localized DYRK kinase Pom1, which inhibits the medially placed mitotic activator Cdr2. Here, Kelkar and Martin show that, upon glucose starvation, microtubules are destabilized in a PKA-dependent manner, leading to the deposition of Pom1 at cell sides where it delays mitosis.

## Graphical abstract

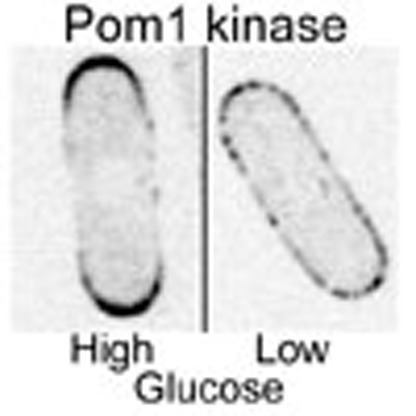

Cell size is a fundamental attribute, critical for cell fitness^1^,^2^,^3^. Cell size is regulated by homoeostatic mechanisms and in response to nutrient availability with cells reducing their target size in conditions of poor nutrients. In rod-shaped fission yeast cells, size control occurs at mitotic commitment. Cyclin-dependent kinase 1 (CDK1) activation for mitotic entry is regulated by the balanced activities of the inhibiting kinase Wee1 and the activating phosphatase Cdc25 (ref. 4). In response to poor nitrogen source, the target of rapamycin (TOR) and mitogen-activated protein kinase (MAPK) stress response pathways modify this balance to advance mitotic commitment and reduce cell size at division^5^,^6^,^7^. When glucose is limiting, cells also reduce their size^8^, though the mechanisms remain largely unknown. Glucose is primarily signalled by a 3′, 5′ cyclic adenosine monophosphate/Protein Kinase A (cAMP/PKA) pathway, which inhibits the transcription of gluconeogenesis and sexual differentiation factors, and also modulates cell cycle progression^9^,^10^,^11^.

In steady-state conditions, cell-intrinsic sizing mechanisms co-ordinate cell growth with division. Recent studies have focused on the DYRK-family kinase Pom1 and its substrate, the Wee1-inhibitory kinase Cdr2 (refs 12, 13). Pom1, which forms concentration gradients from cell poles, restricts Cdr2 localization to the cell middle^14^,^15^ and phosphorylates Cdr2 to inhibit its activation by the Ca^2+^/calmodulin-dependent protein kinase kinase (CaMKK) Ssp1 and delay mitotic commitment^14^,^16^. Pom1 was proposed to co-ordinate growth and division by inhibiting Cdr2 until attainment of sufficient cell length^12^,^13^. Indeed, Pom1 is highly dose-dependent, and its forced localization to cell sides delays mitosis^12^,^13^,^14^. However, it has been unclear where and when it naturally inhibits Cdr2, as medial Pom1 concentration does not substantially vary during cell extension^14^,^17^. In addition, the observation that cells lacking *pom1* remain homeostatic, though at a reduced cell size^18^, has raised questions about whether Pom1 acts as a cell length sensor.

The position of Pom1 gradients is dictated by microtubules, depositing a phosphatase-regulatory complex, Tea1–Tea4, at cell poles^19^,^20^,^21^,^22^. Tea4 associates with a phosphatase 1 catalytic subunit, which dephosphorylates Pom1 to trigger membrane binding^23^,^24^,^25^. Pom1 concentration then decays towards the cell middle through diffusion and autophosphorylation-dependent membrane detachment^25^,^26^. Microtubules form antiparallel bundles nucleated from the nuclear envelope with dynamic plus-ends that grow towards cell poles^27^. Microtubule plus-end dynamics—growth, shrinkage, catastrophe and rescue—are regulated by a host of microtubule-associated proteins (MAPs). These include the +TIP complex Mal3/EB1-Tip1/CLIP-170-Tea2/kinesin, the Alp14/XMAP215 polymerase and the Klp5–6/kinesin-8 (refs 28, 29, 30, 31, 32, 33), which promote microtubule sliding along cell sides and restrict catastrophe events to cell poles for local Tea4 release. Microtubules are maintained in antiparallel bundles by the MAP65/PRC1-family protein Ase1, which localizes to the zones of microtubule overlap, where it recruits the CLIP-170 Associated Protein (CLASP) Cls1/Peg1 (Cls1 below)^34^,^35^,^36^. CLASPs are conserved microtubule stabilizers^37^, initially identified as Cytoplasmic Linker Protein (CLIP)-associated proteins on microtubule plus-end in animal cells^38^. By contrast, the sole fission yeast CLASP does not track microtubule plus-ends, and localizes prominently to zones of antiparallel microtubule overlap, where it is essential for microtubule rescue^34^,^39^.

Here, we describe that Pom1 re-localizes to cell sides and that microtubule dynamics and organization are dramatically altered upon glucose starvation. These findings lead us to uncover a novel PKA-dependent regulation of microtubule dynamics, in which PKA signalling negatively regulates the microtubule rescue factor CLASP to promote microtubule catastrophe, Tea4 delivery and Pom1 re-localization at cell sides, where Pom1 buffers cell size upon glucose starvation.

## Results

### Pka1-dependent re-localization of Pom1 in limited glucose

**Figure 1:**
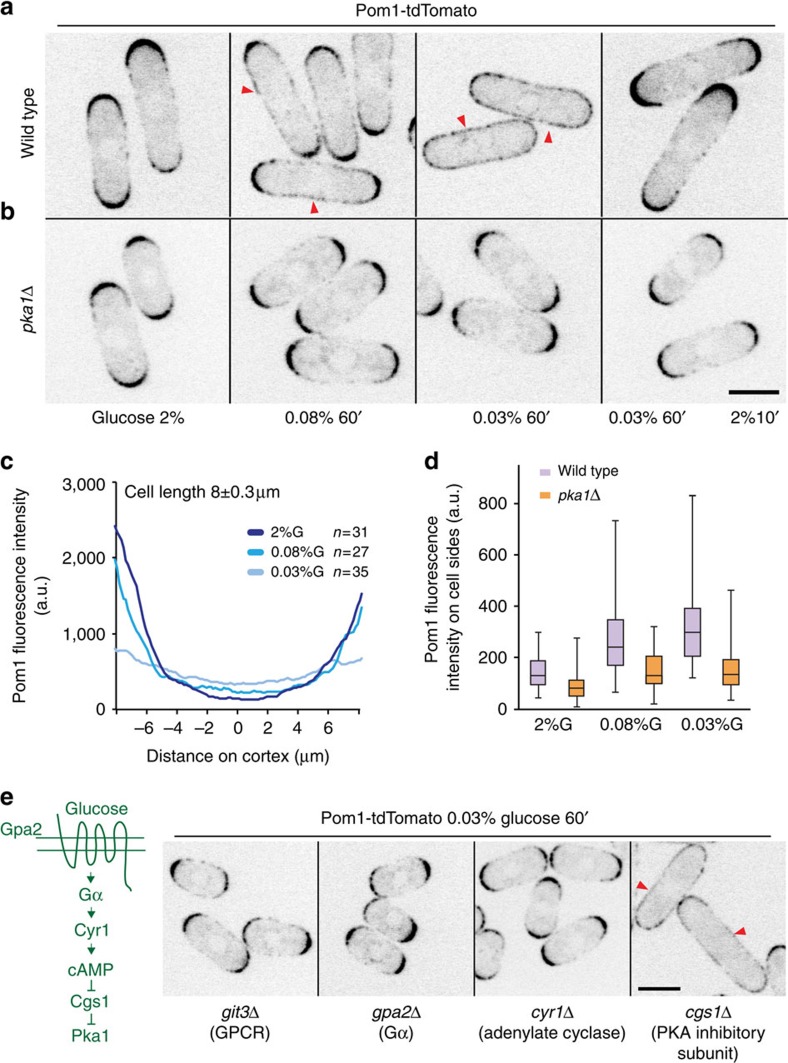
Pka1-dependent reversible re-localization of Pom1 to cell sides upon glucose limitation. (**a**) Sum of five medial spinning disk confocal images taken over 30 s of Pom1-tdTomato in wild-type cells grown in 2% or 0.08% or 0.03% glucose (G) for 1 h. Arrowheads indicate Pom1 presence at cell sides. The last panel shows polar Pom1 after 2% glucose replenishment for 10 min. (**b**) Pom1-tdTomato in *pka1*Δ cells as in **a**. (**c**) Distribution of cortical Pom1 from one cell tip to the other (0=cell middle) in wild-type cells obtained with the Cellophane plugin. Average of 31, 27 and 35 profiles for 2%, 0.08% and 0.03%G, respectively, in 8-μm-long cells. Profiles obtained from other cell lengths are similar. (**d**) Box and whisker plot of cortical Pom1 fluorescence intensity in the middle 2-μm region in both wild-type (*n*=31, 27 and 35) and *pka1*Δ cells (*n*=34, 24 and 42) in 2%, 0.08% and 0.03% glucose respectively. Experiments were performed thrice and quantification of one is shown. (**e**) Left: schematic representation of glucose detection by the PKA pathway. Right: medial spinning disk confocal images of Pom1-tdTomato in mutants of the PKA pathway. Representative images from two independent experiments are shown. Arrowheads indicate Pom1 presence at cell sides. Scale bars are 5 μm.

We serendipitously observed that, in contrast to the polar gradients formed in exponentially growing cells, Pom1 is detected around the medial cortex in saturated cultures (Supplementary Fig. 1A), suggesting that starvation triggers Pom1 re-localization. Depletion of nitrogen or shift from a good to a poor nitrogen source did not modify Pom1 distribution (Supplementary Fig. 1C). By contrast, glucose starvation to levels similar to those measured in the saturated cultures resulted in Pom1 re-localization (Fig. 1a,c,d; Supplementary Fig. 1B). Pom1 was almost uniform around the cell periphery in 0.03% glucose, in which cells grow very little, and was also less confined to cell tips in 0.08% glucose, in which cells proliferate at very similar rates as in 2% glucose^8^. Pom1 re-localization occurred quickly, within 10 min, and reversibly, with no major change in global Pom1 levels as seen by both imaging and western blots (Fig. 1a; Supplementary Fig. 1D). Other stresses, such as temperature (36 °C) or osmotic stress (1 M sorbitol) did not affect Pom1 localization. Thus, Pom1 localization responds to changes in external glucose levels.

Glucose is sensed by a dedicated G-protein-coupled receptor at the plasma membrane, Git3, which signals PKA activation through a G alpha protein, Gpa2, and the adenylate cyclase, Cyr1 (ref. 11). In *pka1*Δ cells grown to saturation, Pom1 remained restricted to cell tips (Supplementary Fig. 1A), indicating that the PKA pathway may be required for Pom1 side-localization upon glucose limitation. Indeed, in *git3*Δ, *gpa2*Δ, *cyr1*Δ and *pka1*Δ cells, Pom1 failed to re-localize after shift to low glucose (Fig. 1b,d,e). By contrast, cells deleted for the regulatory subunit of PKA, *cgs1*, which exhibit constitutive Pka1 activity^40^, displayed normal tip-localized Pom1 in glucose-rich conditions and Pom1 re-localization in low glucose (Fig. 1e). Deletion or mutation in the two other major pathways that transduce information about nutrient availability, MAPK and TOR^41^,^42^, did not prevent Pom1 re-localization in low glucose (Supplementary Fig. 1E,F), though Pom1 re-localization was less severe in *sty1*Δ cells or in cells treated with the MAPK inhibitor SP600125 (ref. 43). Interestingly activation of the MAPK pathway using the hyperactive *wis1*^*DD*^ allele^44^ showed a drastic reduction in global Pom1 levels (Supplementary Fig. 1E). Thus, the cAMP–PKA pathway regulates the re-localization of Pom1 upon glucose limitation with some contribution from the MAPK pathway. In this study, we focused on the role of PKA signalling on Pom1 localization.

We note that the Pom1 substrate Cdr2 also distributed more widely around the cell cortex under limited glucose, in a *pka1*-dependent manner, but remained dependent on Pom1 for exclusion from the cell pole (Supplementary Fig. 1G,H), consistent with Pom1 regulating its cortex-binding^15^.

### Pka1 is active in low glucose to signal Pom1 re-localization

Previous data showed that nuclear-localized Pka1 represses transcription in presence of glucose, with derepression observed upon glucose depletion^45^,^46^,^47^. However, the cortical, fast, reversible dynamics of Pom1 re-localization upon glucose depletion suggest this may not be a transcriptional response.

**Figure 2:**
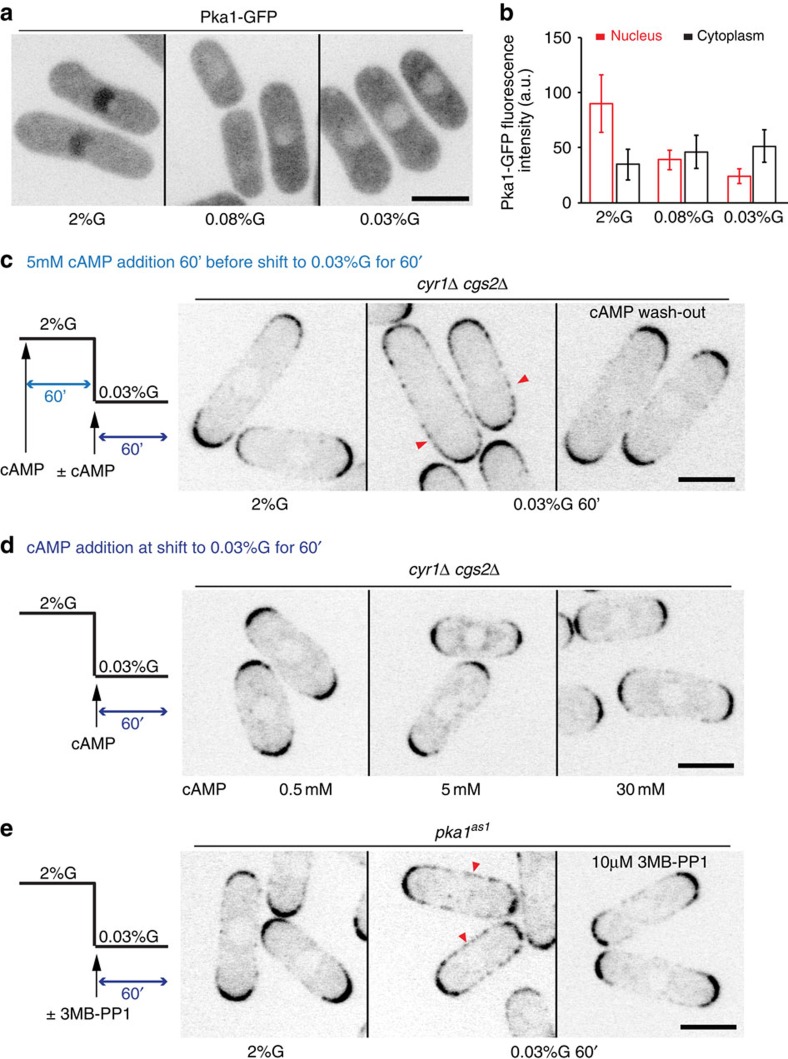
Pka1 is active in low glucose to promote Pom1 side-localization. (**a**) Maximum intensity spinning disk projection of Pka1-GFP in wild-type cells grown in 2% or 0.08% or 0.03% glucose for 1 h. (**b**) Measurement of cytoplasmic and nuclear Pka1-GFP levels in cells as in **a** (*n*>20). Experiments were performed thrice and quantification of one is shown. (**c**) Medial spinning disk confocal section of Pom1-tdTomato in *cyr1*Δ*cgs2*Δ cells incubated with 5 mM cAMP in 2% glucose for 1 h (left) and shifted to 0.03% glucose for 1 h with the same amount of cAMP (middle) or without cAMP (right). Arrowheads indicate Pom1 at cell sides. (**d**) Medial spinning disk confocal section of Pom1-tdTomato in *cyr1*Δ*cgs2*Δ cells grown in 2% glucose and incubated with increasing amounts of cAMP at the time of shift to 0.03% glucose for 1 h. (**e**) Medial spinning disk confocal section of Pom1-tdTomato in *pka1-as1* cells grown in 2% glucose (left) and shifted to 0.03% glucose without (middle) or with 10 μM 3MB-PP1 (right). Arrowheads indicate Pom1 at cell sides. Scale bars are 5 μm. Error bars are s.d. For **c**–**e**, representative images from two independent experiments are shown.

Consistent with previous data^48^,^49^, Pka1 localized to the nucleus in presence of 2% glucose, but was also present in the cytosol, where its concentration increased as it exited the nucleus upon glucose starvation (Fig. 2a,b)^48^. We found that in *cgs1*Δ cells, nuclear and cytosolic levels of Pka1 tagged with green fluorescent protein (GFP) remained constant in all glucose-conditions tested (Supplementary Fig. 2A,B). As these cells showed a wild-type-like Pom1 distribution (Fig. 1e), we conclude that Pka1 may signal Pom1 re-localization from the cytosol, but that an increase in cytosolic Pka1 cannot be the trigger for Pom1 re-localization.

We addressed when Pka1 activity is required in two ways. First, we used cells lacking cAMP, and thus PKA activity, due to deletion of the adenylate cyclase and cAMP phospho-diesterase genes (*cyr1*Δ *cgs2*Δ). As expected, *cyr1*Δ *cgs2*Δ cells failed to re-localize Pom1, but addition of cAMP before and throughout glucose limitation restored PKA activity and yielded a uniform Pom1 localization over the entire cell cortex. However, if cAMP was washed out at the time of shift to low glucose, Pom1 failed to re-localize, suggesting PKA activity is required in low glucose (Fig. 2c; Supplementary Fig. 2D). We note that up to 30 mM cAMP addition only at the time of shift to low glucose was not sufficient to trigger Pom1 re-localization (Fig. 2d). Control *pka1*Δ *cgs2*Δ cells treated the same way retained polar Pom1, confirming that the action of cAMP occurs through PKA activation (Supplementary Fig. 2C,D). Thus, Pka1 is active and required when glucose is limiting to signal Pom1 re-localization.

Second, we constructed an analogue-sensitive Pka1 mutant (*pka1*^*as1*^), whose activity could be selectively inhibited by addition of an ATP-analogue^50^. Untreated *pka1*^*as1*^ cells behaved largely as wild-type cells, re-localizing Pom1 in low glucose (Fig. 2e), though these cells were somewhat shorter than wild type, suggesting Pka1^as1^ is not fully functional (Supplementary Fig. 2E). Prolonged treatment with 10 μM 3MB-PP1 mimicked a *pka1* deletion (Supplementary Fig. 2F). Selective Pka1^as1^ inhibition only upon glucose limitation also blocked Pom1 re-localization, confirming that PKA is active to promote Pom1 side-localization in the glucose-limiting conditions (Fig. 2e). Together, these data suggest that cytosolic Pka1 is active during glucose limitation and signals Pom1 re-localization.

### Mechanism of Pom1 re-localization upon glucose limitation

We considered three possible mechanisms of Pom1 re-localization: first, glucose limitation may lead to Pom1 inactivation, causing its delocalization as observed for a Pom1^KD^ (kinase-dead) allele^25^,^51^; second, changes in membrane composition or potential may increase the intrinsic affinity of Pom1 for the plasma membrane; third, the delivery to cell ends of Tea4, required for Pom1 dephosphorylation and membrane-binding, may be altered. Several lines of evidence—in particular fluorescent recovery after photobleaching (FRAP) analysis, Tea4-dependency and the fact that Pom1 controls cell length in low glucose (see below)—indicated that Pom1 remains active upon glucose limitation, and that glucose limitation only modestly affects the intrinsic affinity of Pom1 for the plasma membrane (see Supplementary Fig. 3 for details).

**Figure 3:**
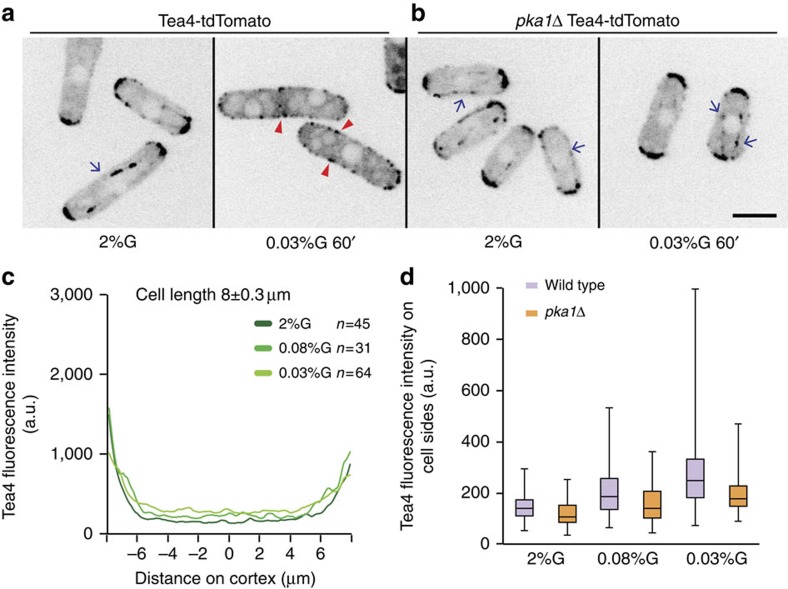
Pka1-dependent reversible Tea4 re-localization around the cell cortex upon glucose limitation. (**a**) Sum of five medial spinning disk confocal images taken over 30 s of Tea4-tdTomato in wild-type cells grown in 2% glucose or 0.03% glucose for 1 h. Arrows indicate Tea4 tracks on microtubules. Arrowheads indicate Tea4 dots at cell sides. (**b**) Localization of Tea4-tdTomato in *pka1*Δ cells as in **a**. (**c**) Distribution of cortical Tea4 from one tip to the other (0=cell middle) in wild-type cells obtained with the Cellophane plugin. Average of 45, 31 and 64 profiles in 2, 0.08 and 0.03% glucose in 8-μm-long cells. Profiles obtained from other cell lengths are similar. (**d**) Box and whisker plot of cortical Tea4 fluorescence intensity in the middle 2 μm region in both wild type (*n*=45, 31 and 64) and *pka1*Δ cells (*n*=71, 66 and 62) in 2%, 0.08% and 0.03% glucose, respectively. Scale bars represent 5 μm. Experiments were performed thrice and quantification of one is shown.

By contrast, the localization of the Pom1 gradient nucleator Tea4 was drastically changed upon glucose limitation (Fig. 3a,c): in 0.08% glucose, there was an increase in the cortical levels of Tea4 in the middle of the cell; in 0.03% glucose, Tea4 dots were present all around the cell periphery and microtubule tracks were not detected. Tea4 re-localization reversibly occurred within 10 min of shift to low glucose and was *pka1*-dependent (Fig. 3b,d). Tea4 was also present on cell sides in saturated cultures (Supplementary Fig. 3D). As localization of Tea4 on cell sides is sufficient to recruit Pom1 (refs 24, 25), we conclude that, upon glucose limitation, Pka1 regulates Tea4 localization, which in turn recruits Pom1.

### Pka1 controls microtubule stability

**Figure 4:**
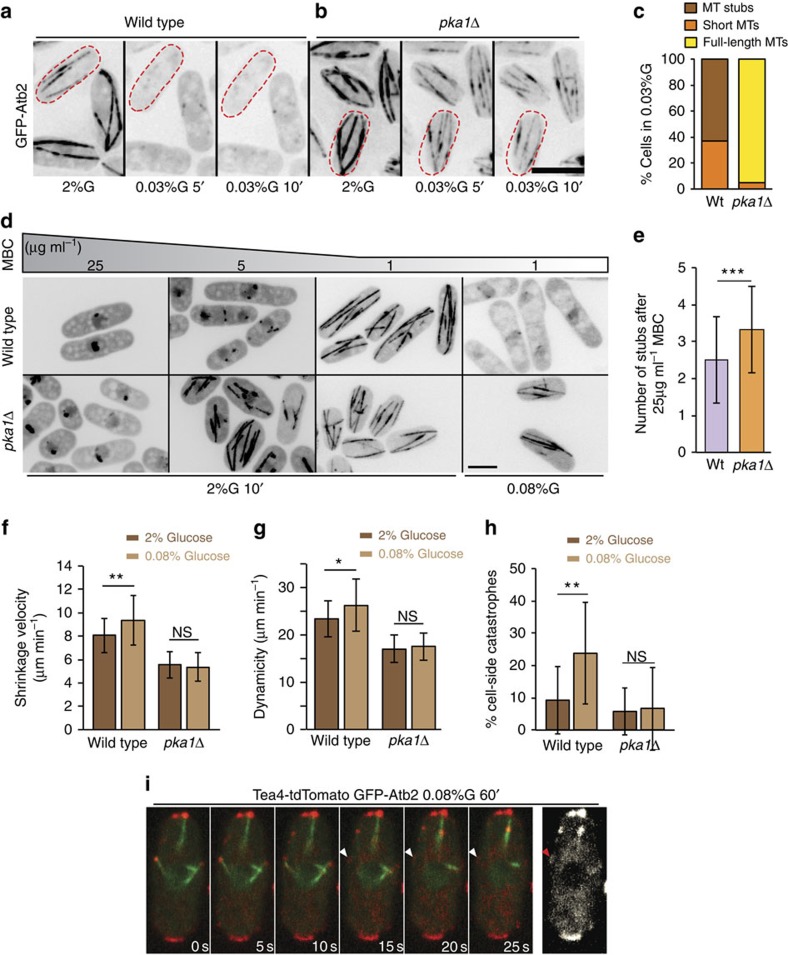
Pka1 negatively regulates microtubule stability. (**a**) Epifluorescence deconvolved maximum intensity projection images of wild-type cells expressing GFP-Atb2 grown in microfluidic chambers in 2% glucose and 5 and 10 min after shift to 0.03% glucose. (**b**) GFP-Atb2 in *pka1*Δ cells as in **a** (2% glucose and 0.03% glucose after 10 min). Representative images from three independent experiments are shown. (**c**) Percentage of wild-type (*n*=68) and *pka1*Δ (*n*=59) cells with indicated microtubule organization in 0.03% glucose for 10 min in microfluidic chambers. (**d**) Maximum projection of spinning disk confocal images of wild-type and *pka1*Δ cells expressing GFP-Atb2 treated with the indicated concentrations of MBC for 10 min in 2% or 0.08% glucose. Representative images from two independent experiments are shown. (**e**) A graph showing the number of microtubule stubs left after 10 min 25 μg ml^−1^ MBC treatment (*n*>45 cells). *P*=0.001. (**f**) Mean microtubule shrinkage velocity in wild-type and *pka1*Δ cells grown with 2% glucose or 0.08% glucose for 1 h (*n*>27 microtubules). (*P*=0.008; *P*=0.56). (**g**) Mean microtubule dynamicity in wild-type and *pka1*Δ cells grown with 2% glucose or 0.08% glucose for 1 h (*n*>27 microtubules; *P*=0.031; *P*=0.54). (**h**) Percentage of microtubule catastrophes occurring at the cell sides in wild-type and *pka1*Δ cells (*n*>100 catastrophe events in >16 cells) grown as in **e**. (*P*=0.002, *P*=0.926). (**i**) Time-lapse imaging of Tea4-tdTomato and GFP-Atb2 in wild-type cells shifted to 0.08% glucose for 1 h acquired on the spinning disk confocal microscope. The first six images are maximum projections of two Z-sections. The last image is a projection of the three time points shown after microtubule catastrophe. Arrowheads indicate Tea4 presence at the lateral cell cortex after microtubule catastrophe. Representative images from four independent experiments are shown. Scale bars represent 5 μm. Error bars are s.d. Statistical significance was derived using student’s *t*-test.

To understand the mechanism of Tea4 re-localization, we probed microtubule behaviour under limited glucose conditions, using flow-chambers. A shift to 0.03% glucose caused a dramatic, rapid, reversible loss of long interphase microtubules in <5 min, with 37% cells exhibiting short dynamic microtubules and 63% displaying only microtubule stubs (stable regions of microtubule overlap; Fig. 4a,c). As medium exchange in the flow-chamber takes about 3 min, the effect occurred within 2 min of glucose deprivation. Mitotic spindles were resistant and kept elongating under these conditions. Short dynamic microtubules were observed in cells kept in 0.03% glucose for up to 20 h, after which microtubules disappeared completely from all cells (Supplementary Movie 1).

In 0.08% glucose, microtubules remained long but were also destabilized. First, they were depolymerized by suboptimal methyl benzimidazol-2-yl-carbamate (MBC) levels (1 μg ml^−1^) that did not affect microtubules in 2% glucose (Fig. 4d). Second, they showed increased shrinkage rate and dynamicity (length change of microtubules in any direction over time), as compared to cells grown in 2% glucose (Fig. 4f,g). Finally, 23.8% of microtubule catastrophes occurred on contact at the lateral cortex (versus only 9.2% in 2% glucose; Fig. 4h). These catastrophes at cell sides led to sustained Tea4 contact and temporary deposition at the lateral cortex (Fig. 4i). Thus, frequent microtubule catastrophes at cell sides may promote Tea4 and thus Pom1 re-localization upon glucose starvation.

Microtubule destabilization after glucose limitation was entirely dependent on Pka1: in *pka1*Δ cells, microtubules remained long when cells were shifted to 0.03% glucose for up to 30 h, after which microtubules disappeared completely as in the wild-type situation (Fig. 4b,c; Supplementary Movie 2); in 0.08% glucose, they were not depolymerized by suboptimal dosage of MBC (Fig. 4d), and dynamic parameters and location of catastrophes at cell sides were not altered as compared with *pka1*Δ cells grown in 2% glucose (Fig. 4f,g); in 2% glucose, microtubules were also significantly more stable in *pka1*Δ than wild-type cells, displaying higher resistance to MBC treatment, and slower shrinkage rate and dynamicity (Fig. 4d–g). Conversely, Pka1 overexpression caused faster microtubule shrinkage rates, higher dynamicity and 30.2% of catastrophes occurring along the lateral cell cortex (Supplementary Fig. 4A; Table 1). As *pka1* overexpression causes cell elongation, we controlled for the effect that cell length may have on microtubule dynamics by using elongated *cdc25-22* cells. In these cells, microtubules showed largely wild-type dynamic parameters, with 20% side catastrophes, a percentage significantly lower than that observed upon *pka1* overexpression (Supplementary Fig. 4A; Table 1). We conclude that Pka1 negatively regulates microtubule stability.

Remarkably, microtubule destabilization was sufficient to trigger both Tea4 and Pom1 side-relocation. Indeed, Tea4 and Pom1 partly re-localized to cell sides in glucose-starved *pka1*Δ cells when microtubules were destabilized either by a 10 min MBC treatment (Supplementary Fig. 4C,D), or by deletion of the +TIP complex (Supplementary Fig. 4E). Similarly, Pom1 localized along cell sides in *pka1*-overexpressing cells even in 2% glucose, but not in *cdc25-22* mutant cells (Supplementary Fig. 4B). Thus, Pka1 promotes microtubule destabilization, which causes Tea4 and thus Pom1 re-localization to cell sides.

### Pka1 regulates microtubule dynamics through CLASP

To dissect the mechanism by which Pka1 destabilizes microtubules, we screened for genetic interactions between *pka1*Δ and mutations in known MAPs in 2% glucose, initially measuring microtubule shrinkage rates. We reasoned that *pka1*Δ would not modify the phenotype of a MAP deletion if this was regulated by PKA, but would show additive effects to other MAP mutants. Although single or even triple deletions of the +TIP complex, in which microtubules are destabilized^28^,^52^,^53^, restored Pom1 side-localization to glucose-starved *pka1*Δ cells (Supplementary Fig. 4E), all deletions showed additive effects with *pka1*Δ for microtubule shrinkage velocities (Table 1). Similarly, deletion of the tumor overexpressed gene (TOG)-domain protein Alp14/XMAP215, shown to accelerate microtubule assembly^32^, was additive with *pka1*Δ. These additive phenotypes indicate that Pka1 does not modulate microtubule stability through these MAPs.

**Figure 5:**
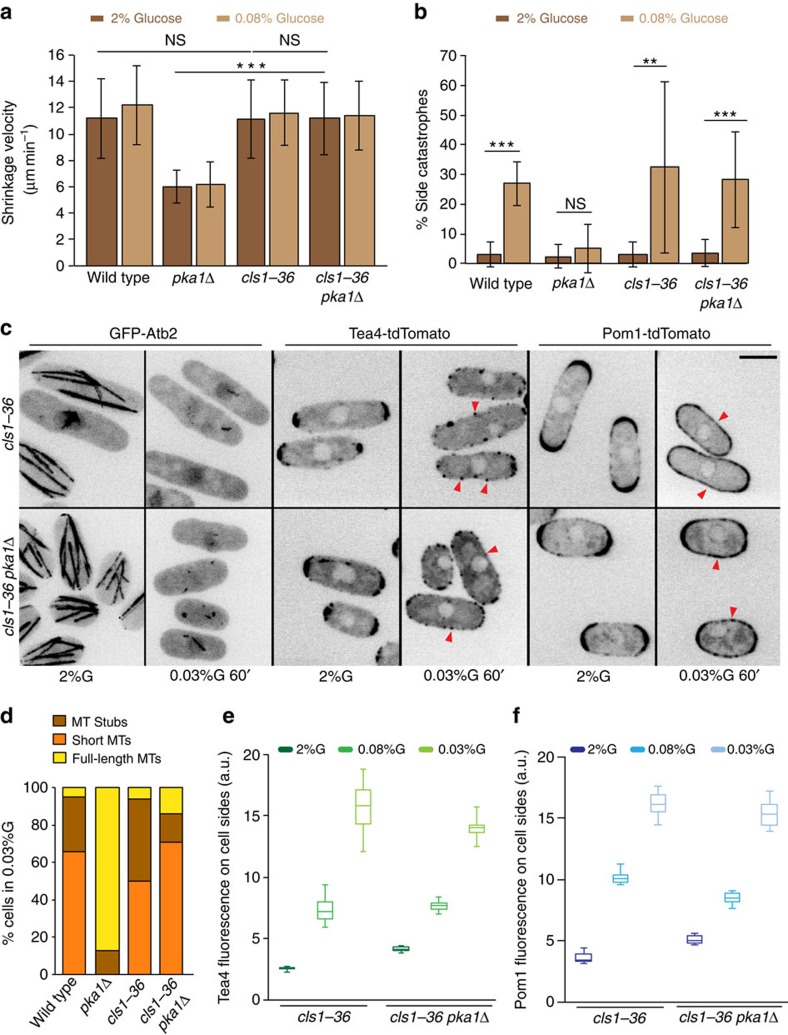
Pka1 regulates microtubule dynamics through CLASP to trigger Tea4 and Pom1 side-localization. (**a**) Mean microtubule shrinkage velocity in wild-type, *pka1*Δ, *cls1-36* and *cls1-36 pka1*Δ strains grown in 2% glucose or 0.08% glucose for 1 h at 36 °C (*n*>15 microtubules; *P*=0.9, *P*=0.93, *P*<10^−9^). (**b**) Percentage of microtubule catastrophes occurring at cell sides in the same strains, grown as in **a** (*n*>70 catastrophe events in >16 cells). Statistical significance is derived using student’s *t*-test. (*P*<10^−8^, *P*=0.46, *P*=0.015, *P*=0.00013). Error bars are s.d. (**c**) Maximum intensity projection of spinning disk images of GFP-Atb2, and sum of five medial spinning disk confocal images taken over 30 s of Tea4-tdTomato and Pom1-tdTomato in *cls1-36* and *cls1-36 pka1*Δ cells grown in 2% glucose at 25 °C and shifted to 36 °C for 1 h in either 2% glucose or 0.03% glucose. Arrowheads indicate Tea4 or Pom1 side-localization. Scale bar represents 5 μm. (**d**) Percentage of wild type (*n*=38), *pka1*Δ (*n*=32), *cls1-36* (*n*=100) and *cls1-36 pka1*Δ (*n*=119) cells showing the indicated microtubule organization after shift to 0.03% glucose for 10 min at 36 °C. (**e**) Box and whisker plot of cortical Tea4 fluorescence intensity in the middle 2 μm region in both *cls1-36* and *cls1-36 pka1*Δ cells at 36 °C with cells grown in 2% glucose at 25 °C and shifted to 36 °C for 1 h in either 2% glucose or 0.08% glucose or 0.03% glucose (*n*>15). (**f**) Box and whisker plot of cortical Pom1 fluorescence intensity in the middle 2-μm region in both *cls1-36* and *cls1-36 pka1*Δ cells at 36 °C with cells grown as in **e** (*n*>19). Experiments were performed thrice and quantification of one is shown.

By contrast, several lines of evidence showed that Pka1 modulates microtubule dynamics through the sole fission yeast CLASP Cls1/Peg1. Consistent with previous observations^34^, inactivation of Cls1, using a published temperature-sensitive mutant *cls1-36* at the restrictive temperature of 36 °C, revealed no or minor effect on microtubule dynamics (Table 1). Surprisingly however, *cls1-36* masked the effect of *pka1*Δ for all microtubule dynamic parameters in both 2% and 0.08% glucose (Fig. 5a). The *cls1-36* mutant also restored frequent side-catastrophes in *pka1*Δ cells in 0.08% glucose (Fig. 5b), and microtubule destabilization in 0.03% glucose (Fig. 5c,d). Thus, in absence of PKA, CLASP modulates microtubule dynamics. We note though that more long microtubules were observed in double *cls1-36 pka1*Δ than single *cls1-36* mutant in 0.03% glucose (Fig. 5d), suggesting PKA may signal through additional MAPs for microtubule destabilization. CLASP inactivation further restored Tea4 and Pom1 side-localization upon glucose starvation in *pka1*Δ cells (Fig. 5c,e,f). Conversely, *cls1* overexpression, which promotes microtubule stabilization^34^, led to restriction of Pom1 at cell tips on glucose limitation, mimicking the *pka1*Δ phenotype (Supplementary Fig. 5). We conclude that Pka1 promotes microtubule destabilization, and thus Tea4 and Pom1 side-localization, by negatively regulating CLASP.

**Figure 6:**
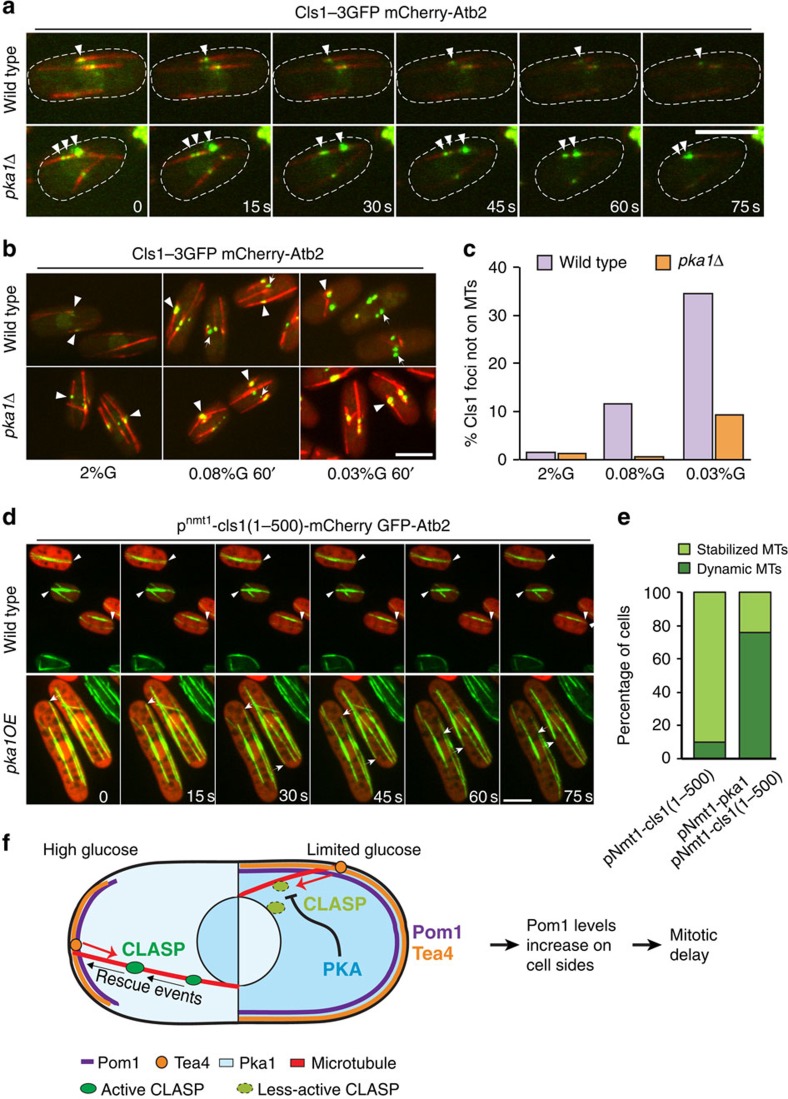
Pka1 promotes microtubule destabilization by negatively regulating Cls1. (**a**) Maximum intensity spinning disk projections showing time-lapse imaging of Cls1-3GFP and mCherry-Atb2 in wild-type and *pka1*Δ cells grown in 2% glucose. Arrowheads highlight Cls1 dots on microtubules. (**b**) Maximum intensity spinning disk projections of Cls1-3GFP and mCherry-Atb2 in wild-type and *pka1*Δ cells grown in 2% glucose or 0.08% glucose or 0.03% glucose for 1 h. Arrowheads indicate Cls1 dots present on microtubule overlaps. Arrows point to Cls1 foci not on microtubules. (**c**) Percentage of Cls1 foci not on microtubules in cells as in **b** (*n*>96). Representative images from three independent experiments and quantification of one are shown. (**d**) Time-lapse imaging of GFP-Atb2 in wild-type and *pka1*-overexpressing (pka1-OE) cells transformed with p^nmt1^-cls1(1–500)-mCherry grown in 2% glucose and induced without thiamine for 14–16 h. Arrowheads indicate stable microtubules in wild type (upper panel) and arrows track microtubule shrinkage events in pka1-OE (bottom panel) cells. Scale bar represents 5 μm. (**e**) Percentage of cells showing dynamic or at least one stabilized microtubule bundle in strains as in **d**. Cells with similar range of Cls1 fluorescence levels were chosen for this analysis (*n*=31 for wild type, *n*=41 for pka1 overexpression). Representative images from two independent experiments and quantification of one are shown. (**f**) Schematic depicting the mechanism of Pom1 side-localization under glucose limitation conditions. The left part of the cell shows the situation in high glucose, when CLASP rescues microtubules allowing them to reach cell ends. The right part of the cell shows the situation in low glucose, when PKA activity antagonizes the microtubule-stabilizer CLASP. Microtubules thus become destabilized and undergo catastrophe at the lateral cortex, depositing Tea4 there. Tea4-mediated dephosphorylation of Pom1 leads to an increase in its levels at cell sides, promoting mitotic delay.

CLASP stabilizes microtubules by binding both microtubules and tubulin directly through its S/R-rich region and N-terminal TOG domains, respectively^34^,^39^. *In vivo*, Cls1 is recruited to zones of microtubule overlaps by the microtubule bundler Ase1 (ref. 34). Dynein-dependent Cls1 localization near microtubule plus-ends was also reported^54^,^55^. Cls1 localization was significantly changed both in low glucose and in *pka1*Δ cells. In *pka1*Δ cells, Cls1 showed increased levels on microtubules and often formed multiple dots on a single microtubule, though global Cls1 levels were only modestly changed (Fig. 6a, Supplementary Fig. 6A–C). In low glucose, Cls1 also showed enhanced local levels and formed strong foci near the cell middle (Fig. 6b). Whereas most of these foci localized on microtubule bundles in *pka1*Δ cells, 12–35% were at sites lacking microtubules in wild-type cells (Fig. 6c). These foci that do not contain microtubules may represent less active or inactive Cls1 in agreement with the less stable microtubule bundles in these conditions. We were unable to test for the presence of Ase1 in these foci, as colocalization experiments using Ase1-mCherry and Cls1-3GFP showed synthetic effects. However, deletion of neither Ase1 nor the dynein Dhc1 masked the effect of *pka1*Δ on microtubule dynamics, suggesting Pka1 does not regulate Cls1 solely through these targeting factors (Table 1). Thus PKA may render Cls1 less active for microtubule rescue, leading to complete loss of the microtubule bundle. In addition, we found that Pka1 overexpression was able to mitigate the Cls1 TOG domains-dependent microtubule stabilization (Fig. 6d,e), suggesting Pka1 reduces the activity of Cls1 in microtubule stabilization.

### Pom1 re-localization buffers against excess cell shortening

We examined the physiological consequences of Pom1 re-localization in low glucose, by examining cell length at division as a proxy of cell cycle length^56^. We measured cell length at division in the standard conditions of 2% glucose and in 0.08% glucose, in which cells show reliable growth and division patterns, though at shorter size^8^ (Table 2).

Pka1 plays an ill-understood function in cell cycle progression. Indeed, *pka1*Δ cells are short, and *pka1* overexpression yields long cells, suggesting Pka1 delays the cell cycle^9^,^10^. Previous work placed Pka1 upstream of MAPK signalling^57^,^58^. Consistently, in 2% glucose, deletion of the MAPK Sty1 was largely epistatic over Pka1, with *sty1*Δ *pka1*Δ double mutants dividing at a length similar to *sty1*Δ single mutants; and *pom1*Δ and *pka1*Δ were additive, with double mutants dividing at shorter size than either single mutant. By contrast, in 0.08% glucose, *sty1*Δ *pka1*Δ double mutants were significantly shorter than *sty1*Δ single mutants, suggesting that in these conditions Pka1 plays an additional role, not upstream of Sty1; in addition, *pom1*Δ *pka1*Δ double mutant divided at the same length as *pom1*Δ single mutant, suggesting Pom1 is largely epistatic over Pka1 in these conditions. We conclude that Pka1 regulates both MAPK and Pom1 pathways, with contributions depending on glucose conditions.

To better understand the changes in cell size on glucose limitation, we devised an adaptation index representing the per cent difference in cell lengths between 2% and 0.08% glucose. Wild-type cells reduced in length by about 12%. As is the case on nitrogen limitation^5^,^6^,^7^, MAPK likely promotes mitotic entry on glucose limitation, as *sty1*Δ cells did not shorten in 0.08% glucose. Instead, these cells became longer, yielding a negative adaptation index. Remarkably, this negative adaptation was abolished by *pom1* deletion. Consistently, *pom1* deletion or inactivation caused hyper-adaptation with cells loosing 25% of their length in low glucose, suggesting Pom1 plays a comparatively more important role to delay mitosis in glucose-limiting conditions.

We confirmed that the newly recruited, membrane-associated Pom1 activity delays mitosis through Cdr2 regulation, as in cells grown in glucose-rich medium. First, *pom1*^*KD*^ cells bearing a kinase-dead allele were indistinguishable from *pom1*Δ, indicating Pom1 kinase activity is required. Second, *tea4* mutant cells, in which Pom1 is largely cytosolic^25^, showed hyper-adaption to glucose limitation like *pom1*Δ, indicating that Pom1 signals from the plasma membrane. Third, mutation of 6 Pom1 autophosphorylation sites in *pom1*^*6A*^, previously shown to delocalize active Pom1^6A^ around the cell cortex even in rich medium^25^, severely dampened cell length adaption to low glucose. Fourth, *cdr2*Δ was epistatic to *pom1*Δ under limiting glucose, like in rich medium, and also showed dampened adaptation. Finally, we found that the *cdr2*^*T166A*^ allele, carrying a mutation of the activation loop preventing phosphorylation by the activating kinase Ssp1 (ref. 16), showed hyper-adaption to glucose limitation, similar to *pom1*Δ. This is consistent with the proposed model that Pom1 blocks activation of Cdr2 by Ssp1 (ref. 16). However, mutation of the C-terminal Pom1 phosphorylation sites in *cdr2*^*S755A–758A*^, through which Pom1 was proposed to inhibit Cdr2 activity^14^,^16^, was epistatic to *pom1*Δ in high, but not low glucose, and did not show hyper-adaption to glucose limitation, suggesting other modes of Cdr2 regulation by Pom1 exist.

Together these data suggest that the higher levels of Pom1 along cell sides in low glucose conditions impose a stronger inhibition on Cdr2, balancing the mitosis-promoting effect of the MAPK pathway.

## Discussion

We report here two physiological changes in cells grown in limiting glucose levels, a condition that cells may routinely face in the wild. First, the mitotic inhibitor Pom1 re-localizes to cell sides upon glucose limitation, where it delays division. Second, microtubule organization and dynamics are severely altered. Both changes rely on PKA signalling negatively regulating the activity of the microtubule rescue factor CLASP. Thus, when glucose is limiting, microtubules are destabilized and undergo catastrophe at cell sides, where they transiently deposit the type I phosphatase-regulatory subunit Tea4. This in turn promotes Pom1 membrane binding at cell sides, which delays cell division (Fig. 6f).

PKA signals microtubule destabilization. Indeed, microtubules are stabilized and insensitive to glucose levels in cells deficient in the cAMP/PKA signalling cascade, whereas Pka1 overexpression promotes microtubule destabilization. Previous investigation of PKA had shown it localizes to the nucleus in glucose-rich conditions^48^, where is serves to repress transcription^46^,^47^. By contrast, cytosolic PKA likely controls microtubule dynamics. Indeed, the PKA-dependent destabilization of microtubules on starvation is rapid (occurring within 2 min) and reversible, and thus unlikely to be due to a transcriptional response^59^. We further showed that Pka1 is active in glucose-limiting conditions and that its activity is required to signal Pom1 re-localization. These data are consistent with a cytosolic microtubule-destabilizing PKA activity in glucose-limiting conditions.

We note though that PKA is unlikely to be the sensor of glucose limitation to trigger microtubule destabilization, because *cgs1*Δ mutants and *cls1-36 pka1*Δ double mutants, which have constitutive and no PKA activity, respectively, still re-localize Pom1 to cell sides specifically upon glucose limitation. Thus, while PKA activity is necessary for microtubule destabilization, it is not sufficient for it. We deduce that an additional pathway signals microtubule destabilization and Pom1 re-localization on glucose depletion. Because of the alterations in Pom1 levels and localization observed in *sty1* and *wis1* mutants (Supplementary Fig. 1E) and given the genetic relationships of *pka1* and *sty1* (refs 57, 58), the stress MAPK pathway may be a prime candidate for this additional signal.

CLASP-dependent microtubule stabilization is a likely target of the PKA signal. *In vitro*, CLASP promotes rescue and suppresses catastrophes by recruiting tubulin dimers to the microtubules^39^. In fission yeast, CLASP is recruited to zones of microtubule overlap by Ase1, where it promotes microtubule rescue and the maintenance of the microtubule bundle, but does not appear to affect microtubule plus end dynamics (ref. 34 and our data). This is in contrast to the situation in metazoan cells, where CLASP is recruited to microtubule plus-ends by CLIP-170 and protects them from catastrophe^38^, or in plant cells, where CLASP promotes microtubule growth at cell edges^60^. We now show that, in *pka1*Δ cells, CLASP loss-of-function restores microtubule depolymerization rate and catastrophes at cell sides, as well as Tea4 and Pom1 relocation, in low glucose. We conclude that CLASP also promotes microtubule growth persistence in fission yeast, but that this activity is normally inhibited by PKA signalling. Thus, tuning of CLASP activity underlies distinct microtubule organization.

PKA signalling likely antagonizes the microtubule-stabilizing function of CLASP. In glucose-starved wild-type cells, a significant number of Cls1 foci do not contain microtubules. We interpret these as sites of inactive Cls1, unable to promote microtubule rescue, from which the microtubule bundle has been lost. Contrary to this, in *pka1*Δ cells, most Cls1 foci contain microtubules, suggesting Cls1 is functional for microtubule rescue. In addition, Pka1 overexpression antagonizes the microtubule-stabilizing effect of the CLASP TOG domains, which directly bind tubulin dimers. PKA signalling may also antagonize CLASP microtubule binding, as CLASP localization on microtubule overlaps is enhanced in *pka1*Δ cells. We suggest that, similar to *in vitro* observations^39^, in absence of PKA, direct binding by a few CLASP molecules near a microtubule plus end leads to rescue events promoting further growth of microtubules contacting cell sides.

We note however that CLASP is unlikely to be a direct PKA substrate, because mutation of five predicted PKA phosphorylated sites in Cls1 N-terminus did not alter Cls1’s ability to stabilize microtubules *in vivo*, and immuno-precipitated Pka1-GFP failed to phosphorylate Cls1 fragments *in vitro*. PKA may also indirectly modulate microtubules by regulating the cell energy levels. Indeed, recent data in the budding yeast has shown that cells lacking PKA activity exhibit higher ATP stores^61^. The complete loss of microtubules we observe after long-term starvation in 0.03% glucose may reflect a loss of cellular energy, which indeed happens later in the *pka1* deleted cells. Thus, PKA may regulate microtubule dynamics in direct and indirect ways.

The cAMP/PKA pathway modulates the cell cycle^9^,^10^, and we show its effect depends on glucose levels. When glucose is in excess, PKA acts principally through the stress MAPK pathway, by ways that remain to be characterized^57^,^58^. By contrast, when glucose is limiting, PKA signals through Pom1 in addition, by modifying its localization. The predicted net effect is an alteration of the balance between Cdc25 and Wee1 activities in favour of Wee1, to delay mitotic commitment. This counterbalances the effect of MAPK activation helping cells to attain a critical size.

Mitotic entry is exquisitely sensitive to Pom1 dosage, but natural changes in Pom1 amounts at the cell middle in steady-state growing conditions had so far not been observed^12^,^13^,^14^,^17^,^25^. We now show that modulation of Pom1 concentration at the medial cortex is a physiological cellular response to glucose levels. Pom1 side-enrichment in 0.08% glucose has important effect on cell size at division by regulating Cdr2. This is especially noticeable in absence of the MAPK Sty1, when Pom1 causes an increase in cell size. By contrast, these localization changes do not have major effect on medial division, consistent with the lower sensitivity of division site placement on Pom1 levels^14^,^24^. Thus, one important function of the mitotic inhibitor Pom1 is to buffer cell size during glucose starvation. Future work should reveal whether this is due to the modification of a homoeostatic sizer system.

## Methods

### Strain construction, media and growth conditions

All *Schizosaccharomyces pombe* strains used are listed in Supplementary Table 1. All plasmids used are listed in Supplementary Table 2. Strains were made by either tetrad dissection or random spore analysis and replication on appropriate antibiotic plates. Gene tagging and deletions were done using a PCR-based approach and confirmed by diagnostic PCR^62^. Transformations were done using the lithium–acetate–dimethylsulphoxide method. Pka1-overexpressing strains were obtained as follows: For KanMX-3nmt1-GFP-Pka1 strain (Supplementary Fig. 4A,B), the first 547 bp of Pka1-open reading frame (ORF) and the last 500 bp of Pka1-promoter were cloned in a pFA6a-KanMX6-P3nmt1-GFP plasmid using BamHI/SalI sites in frame after GFP and SacII/EcoRV sites before the kanMX cassette, respectively. The plasmid was cut with SacII/SalI to excise the cassette with the homology and transformed in appropriate yeast strains, wherein the integration was checked by diagnostic PCR. For NatMX-3nmt1-Pka1 strain (Fig. 6d lower panel), the kanMX6 cassette from pFA6a-kanMX6-P3nmt1 was replaced by the natMX cassette, excised from pFA6a-natMX6 with EcoRV/BglII, followed by the same strategy as above.

To generate the *pka1-as1* strain, the ORF of Pka1 and 618 bp of the 3′UTR were cloned in a small plasmid using BamHI/SalI sites and the gatekeeper residue aa278 (Met) was mutated to (Gly) M278G^63^ by site-directed mutagenesis and checked by sequencing. The cassette was then excised using the same enzymes and transformed into a pka1: ura4+ strain which still retains the first 200 bp of the Pka1 ORF. Colonies were selected on 5′FOA plates and correct integration was checked by diagnostic PCR.

Cells were grown in standard Edinburgh minimal media (EMM) with appropriate supplements adenine, leucine and uracil when required. For glucose limitation assays, cells were grown in 2% glucose to mid-log, washed three times in either 0.08% glucose or 0.03% glucose and then incubated in the same medium before imaging. For measurement of glucose in the medium (Supplementary Fig. 1B), cells were grown in EMM-2% glucose to saturation and glucose concentration in the medium was measured using the Glucose (HK) Assay Kit (Sigma) at the indicated time points. For nitrogen depletion experiment, cells were first grown in EMM medium containing nitrogen and then shifted to medium lacking nitrogen for the indicated time points.

Adenosine 3′,5′-cyclic-monophosphate (cAMP) (Sigma A9501), was used at final concentrations as indicated from a stock of 30 mM dissolved in the same medium as used for imaging. 4-Amino-1-tert-butyl-3- (3-methyl benzyl) pyrazolo (3,4-d pyrimidine) 3MB-PP1 (Toronto Research Chemicals Inc.) was added at final concentrations as indicated from a stock of either 5 or 1 mM in methanol. Methanol addition had no effect on cell length or Pom1 localization. In Fig. 4a,b cells were first grown in EMM with proper supplements, containing 2% glucose to mid-log phase. They were diluted to an optical density (O.D) of 0.1–0.2, and 100 μl of cells were loaded onto ONIX microfluidic chambers (Y04C plates, CellAsic). After loading, the medium flux was kept constant at 2 psi, at which the chamber refresh time is just above 3 min (CellAsic Y04C specifications). These cells were first allowed to adapt to the chambers by letting them grow for 3–4 h before imaging. GFP-Atb2 was imaged in these cells in 2% glucose every 5 min for 15 min, followed by a change in medium to 0.03%G-imaged every 5 min for 30 min.

Methyl-benzidazole-2-yl-carbamate (Sigma, St Louis, MO) was used at a final concentration as indicated, from a stock of 2.5 mg ml^−1^ in dimethylsulphoxide. To induce the expression of Pka1, from the nmt1 promoter cells were first grown in a medium containing 5 μg ml^−1^ thiamine from a 2,000 × stock, to repress the promoter, washed three times in the same medium without thiamine and incubated for 18–20 h before imaging. All temperature-sensitive strains were first grown at their permissive temperature of 25 °C then shifted to their restrictive temperature of 36 °C in either 2%G, 0.08% glucose or 0.03% glucose and imaged using a heated objective at 36 °C, except the *cdc25-22* which was grown at the semi-permissive temperature of 30 °C. All other strains were grown at 30 °C and imaged at room temperature (around 23–25 °C) unless stated otherwise. For cell-length measurements (Table 2) all strains used were prototrophs and grown in EMM, except for *cdr2*^*S755A–758A*^*, pom1*Δ *cdr2*^*S755A–758A*^ and corresponding control strains, which were grown in EMM-adenine, leucine and uracil, with either 2% glucose or 0.08% glucose to mid-log before imaging. Calcofluor (Sigma) was added at a final concentration of 5 μg ml^−1^ from a 200 × stock solution. Hoechst (Sigma) was added at a concentration of 1 μg ml^−1^ for about 15–20 min before imaging.

### Microscopy

Microscopy was performed on live cells, either on a spinning disk confocal microscope or on a DeltaVision epifluorescence system. Spinning disk microscopy was carried out using a Leica DMI4000B inverted microscope equipped with HCX PL APO X100/1.46 (numerical aperture (NA)) oil objective and a PerkinElmer Ultraview Confocal system (including a Yokagawa CSU22 real-time confocal scanning head, and solid-state laser and a cooled 14-bit frame transfer EMCCD C9100-50 camera)^64^. Stacks of z-series confocal sections were acquired at 0.3 μm intervals with the Volocity software. For quantifications of Pom1 and Tea4 using Cellophane^14^, 5 images were taken over 30 s and summed at 1 s exposure time, binning 1. To measure microtubule dynamics (Table 1), images were acquired on the spinning-disk microscope as 3 medial Z-stacks at an interval of 0.5 μm every 5 s for 10 min and analysis was done on maximum projections. FRAP experiments were performed on the spinning disk using the PerkinElmer photokinesis module. One full tip was bleached and post-bleach images were acquired at 5 s intervals for the first 60 s followed by 10 s intervals for the next 140 s and finally 30 s intervals for the last 600 s to minimize bleaching during image acquisition^25^. For Fig. 4a,b, wide-field fluorescence microscopy was performed on a DeltaVision platform (Applied Precision) composed of a customized Olympus IX-71 inverted microscope and a UPlan Apo × 100/1.4 NA oil objective, a CoolSNAP HQ2 camera (Photometrics), and an Insight SSI 7 color combined unit illuminator. 7 Z-stacks were acquired at an interval of 0.6 μm and a maximum projection was taken and deconvolved. For cell-length measurements (Table 2), images of calcofluor-stained cells were taken using the same setup with a Plan Apo × 60/1.42 NA objective. Figures were prepared with ImageJ64 and Adobe Photoshop CS5 and assembled using Adobe Illustrator CS5.

### Biochemistry

For western blots protein extracts were prepared in CXS buffer (50 mM HEPES, pH 7.0, 20 mM KCl, 1 mM MgCl, 2 mM EDTA, pH 7.5 and protease inhibitor cocktail) from cells grown in EMM with appropriate glucose concentrations, using glass beads and a bead beater and 25 μg of protein was loaded and resolved on a SDS–polyacrylamide gel electrophoresis gel. Primary antibodies used were anti-GFP (Roche) and anti-tubulin (TAT1), secondary antibody was anti-mouse-horseradish peroxidase (Promega) used at a concentration of 1:3,000.

### Image analysis

Image analysis for cortical quantification of Pom1, Cdr2 and Tea4 in Figs 1c,d and 3c,d and Supplementary Figs 1G and 4D was done using the manual mode of the ImageJ plugin-Cellophane^14^. A line was drawn around the cortex to measure the fluorescence intensity and the plugin also extracts values of the cytoplasmic signal and the background. The profiles were aligned and further analysed using the R-software. The profiles depicted span from one cell tip to the other. The mean fluorescence intensity measured in the medial 2 μm region from all the cells was used for the bar and whisker plots as shown in the panels. Cortical Pom1 and Tea4 measurements in Fig. 5e,f were done manually using the segmented line tool plugin of ImageJ on images taken as for Cellophane analysis. A 3-pixel line was drawn along the cell periphery from one tip to the other for 20 cells and the fluorescence intensities were obtained using the Plot profile tool. The intensities were corrected for background obtained from profiles of the same line dragged just outside the cell and the corrected intensities in the middle of each cell was selected. The mean fluorescence intensity of the medial 2 μm region of 20 cells was used for the bar and whisker plots shown.

To calculate global fluorescence intensities (Supplementary Figs 1D and 6A), a sum projection of 14 z-stacks on the spinning disk confocal microscope was done for each cell. The polygon tool of ImageJ was used to draw a line manually around the cell periphery and the mean fluorescence intensity inside the cell was obtained using the Measure tool. The fluorescence intensity was corrected for background intensity taken just outside the cell and for cells not expressing GFP or tdTomato. To measure the ratio of cls1 over microtubules in Supplementary Fig. 6B, a sum intensity projection of the 14 z-stacks was done on individual cells imaged on the spinning disk. A 5-pixel line was drawn along a microtubule, using the Straight Line tool in ImageJ and the intensities were obtained using the Plot profile tool. The maximum intensity for each cell was obtained for >27 cells for both the green fluorescent protein (GFP) and red fluorescent protein (RFP) channels and a ratio of GFP/RFP was calculated. The mean of the ratios is shown on the graph.

FRAP quantifications were done as follows, the mean fluorescence intensities were measured over time in three regions: (1) the photo-bleached region (tip), (2) the background and (3) another non-bleached cell. For each time point, the intensities of the bleached region and that of the non-bleached cell were adjusted by subtracting background signal. To correct for loss of signal due to imaging, the adjusted bleached region intensity was then divided by the adjusted intensity of the other cell. For each experiment, all values were normalized so that the prephoto-bleaching value equals 1 (ref. 65). The data were plotted and fit to an exponential and t1/2 derived using the Igor Pro 6 software (Wavemetrics).

For measurements of microtubule dynamics, the ImageJ Manual Tracking plugin was used to measure the growth and shrinkage velocities. Microtubule dynamicity was calculated as follows^66^,^67^. Briefly growth and shrinkage rates were calculated as the change in microtubule length (growth or shrinkage) in time and averaged. Frequency of catastrophe and rescue were measured on a per microtubule basis over 5 min of imaging, and averaged. Dynamicity was calculated by using the following formula^66^: dynamicity=((growth rate/frequency catastrophe)+(shrinkage rate/frequency rescue)) × (frequency catastrophe+frequency rescue).

For cell length measurements done on calcofluor-stained cells in Supplementary Fig. 2C and Table 2, a line was drawn manually across the length of septated cells from the middle of one tip to the other and the length measured using the ImageJ Measure tool. To derive the adaptation index (Table 2) the difference between the length in 2% glucose and 0.08% glucose was normalized to the length in 2% glucose. All the graphs represent mean values and the error bars indicate s.d. except for Figs 1d, 3d and 5e,f and Supplementary Fig. 4D where box and whisker plots are shown. The middle line in the box represents the median value. The upper and lower edges of the box show the upper and lower quartile respectively. The whiskers/bars represent the greatest and the lowest value in the given data set.

### Statistical analysis

Student’s *t*-test was used throughout to test statistical significance of differences in pairwise comparisons. The level of significance is shown with asterisks with * indicating *P*<0.05, **P<0.01 and ***P<0.001. NS indicates not significant (*P*>0.05). The exact *P* values are stated in the figure legends.

Sample size (*n*) in each experiment was chosen as a number sufficient for statistical analysis, but manageable in terms of analysis time. The exact sample size is indicated in the figure and/or figure legend. No samples were excluded from the analysis and no randomization was used.

## Additional information

**How to cite this article:** Kelkar, M. & Martin, S. G. PKA antagonizes CLASP-dependent microtubule stabilization to re-localize Pom1 and buffer cell size upon glucose limitation. *Nat. Commun.* 6:8445 doi: 10.1038/ncomms9445 (2015).

## Supplementary Material

Supplementary Information Supplementary figures 1-6, Supplementary Tables 1-2 and Supplementary References

Supplementary Movie 1 Microtubules in wild-type cells remain short and dynamic up to 20h in 0.03% glucose after which they disappear completely. Wildtype cells expressing GFP-Atb2 were grown first in 2% glucose and shifted to 0.03% glucose in microfluidic chambers. Movies are maximum projections of widefield deconvolved epifluorescence images acquired at 0.8μm interval every hour for 40h. Scale bar is 5 μm. Time is indicated in hours.

Supplementary Movie 2 Microtubules in *pka*1Δ cells remain long up to 30h in 0.03% glucose after which they disappear completely like in wild-type cells. *pka*1Δ cells expressing GFP-Atb2 were grown first in 2% glucose and shifted to 0.03% glucose in microfluidic chambers. Movies are maximum projections of widefield deconvolved epifluorescence images acquired at 0.8μm interval every hour for 40h. Scale bar is 5 μm. Time is indicated in hours.

## Figures and Tables

**Table 1:** Microtubule dynamic parameters.

	**%G**	**Genotype**	**Shrinkage rate (μm min**^−1^)	**Growth rate (μm min**^−1^)	**Frequency of catastrophes (min**^−1^)	**Frequency of rescues (min**^−1^)	**Dynamicity (μm min**^−1^)	**% Side catastrophes**
25 °C	2	WT	8.06±1.47	3.3±0.57	0.53±0.13	0.51±0.14	23.4±3.84	9.17±10.5
	0.08	WT	9.34±2.1	2.9±0.83	0.63±0.28	0.61±0.2	26.2±5.5	23.80±15.7
	2	*pka1*Δ	5.55±1.13	2.95±0.56	0.53±0.13	0.6±0.19	17.06±2.9	5.80±7.3
	0.08	*pka1*Δ	5.36±1.23	3.06±0.70	0.36±0.12	0.36±0.14	17.53±2.9	6.60±12.9
								
36 °C	2	WT	11.2±3.04	4.6±1.37	0.59±0.2	0.58±0.15	31.83±8.25	3.07±4.2
	0.08	WT	12.2±3.0					26.8±7.3
	2	*pka1*Δ	6.03±1.25	3.8±0.68	0.41±0.09	0.47±0.11	19.63±2.14	2.4±3.9
	0.08	*pka1*Δ	6.19±1.7					4.9±8.1
	2	*cls1-36*	11.13±2.9	4.45±0.8	0.47±0.16	0.44±0.15	31.89±6.16	3.1±4.3
	0.08	*cls1-36*	11.7±2.5					32.4±28.8
	2	*cls1-36 pka1*Δ	11.19±2.7	4.09±0.9	0.5±0.14	0.48±0.12	31.33±6.59	3.58±4.58
	0.08	*cls1-36 pka1*Δ	11.4±2.6					28.1±16.05
								
25 °C	2	*Pka1-OE*	13.1±2.1	3.59±0.45	0.71±0.15	0.7±0.17	32.1±8.1	30.2±24.3
		*cdc25-22*	8.35±1.3	3.93±0.98	0.37±0.2	0.39±0.17	25.2±4.2	20.1±16.9
		*mal3*Δ	6.09±1.45					
		*mal3*Δ *pka1*Δ	4.5±0.85					
		*tea2*Δ	10.09±2.23					
		*tea2*Δ *pka1*Δ	6.98±1.26					
		*tip1*Δ	7.74±1.03					
		*tip1*Δ *pka1*Δ	5.98±0.83					
		*tip1*Δ *tea2*Δ *mal3*Δ	8.4±2.0					
		*tip1*Δ *tea2*Δ *mal3*Δ *pka1*Δ	5.0±0.18					
		*alp14*Δ	4.7±0.58					
		*alp14*Δ *pka1*Δ	3.7±0.58					
		*ase1*Δ	8.3±1.67					
		*ase1*Δ *pka1*Δ	5.7±1.24					
		*dhc1*Δ	8.0±1.23					
		*dhc1*Δ*pka1*Δ	4.62±1.12					

WT, wild type.

**Table 2:** Mean cell length at division.

**Genotype**	**Mean cell length at division**		**Adaptation index**	***P***-**value**
	**2%G**	**0.08%G**		
WT	13.9±1.33	12.2±1.57	12.23	>10^−14^
*pka1*Δ	11.14±1.9	10.94±3.03	1.79	0.561090743
*sty1*Δ	23.04±2.61	24.6±5.4	−6.77	0.0002
*sty1*Δ *pka1*Δ	22.7±3.1	21.2±3.2	6.6	>10^−7^
*pom1*Δ	11.58±1.7	8.69±1.26	24.95	>10^−49^
*pom1*Δ *pka1*Δ	9.07±1.77	8.64±2.17	4.74	0.116039099
*pom1*Δ *sty1*Δ	17.4±1.99	16.6±2.08	4.59	0.0048
*cdr2*Δ	22.25±1.86	21.24±3.9	4.5	>10^−8^
*pom1*Δ *cdr2*Δ	21.8±3.02	20.5±2.95	6.3	>10^−6^
*pom1* ^*6A*^	19.38±1.38	18±3.5	7.12	>10^−5^
*pom1* ^*KD*^	11.5±1.5	8.2±1.7	28.69	>10^−34^
*tea4*Δ	15.1±1.01	9.6±0.9	36.5	>10^−82^
*tea4* ^RVXF*^	15.1±0.9	10.5±1.1	30.67	>10^−60^
*cdr2* ^*T166A*^	18.22±1.57	14.34±1.7	21.42	>10^−37^
WT *(YSM2224)*	13.08±1.55	11.69±2.31	10.6	0.0003
*pom1*Δ *(YSM2229)*	11.25±1.38	8.34±1.46	25.8	>10^−18^
*cdr2*^*S755A–758A*^ *(YSM2226)*	12.46±1.08	11.03±2.16	11.47	>10^−7^
*pom1*Δ *cdr2*^*S755A–758A*^ *(YSM2234)*	12.11±2.24	8.52±1.24	20.71	>10^−31^

WT, wild type.

The *P* value was calculated by using the student’s *t*-test comparing the cell length at division in 2% glucose versus 0.08% glucose for the same strain. A *P* value <0.05 was considered to indicate a significant difference.

All strains used are prototroph, except for the bottom four (YSM2224, YSM2226, YSM2229 and YSM2234), which are auxotroph for leucine and uracil.
