# Supplementary material for: PKA antagonizes CLASP-dependent microtubule stabilization to re-localize Pom1 and buffer cell size upon glucose limitation
Source: Nat Commun. 2015 Oct 7;6:8445. doi: 10.1038/ncomms9445 (PMC4618306; doi:10.1038/ncomms9445)
Supplement: Supplementary Information — Supplementary figures 1-6, Supplementary Tables 1-2 and Supplementary References [file ncomms9445-s1.pdf]

Figure S1

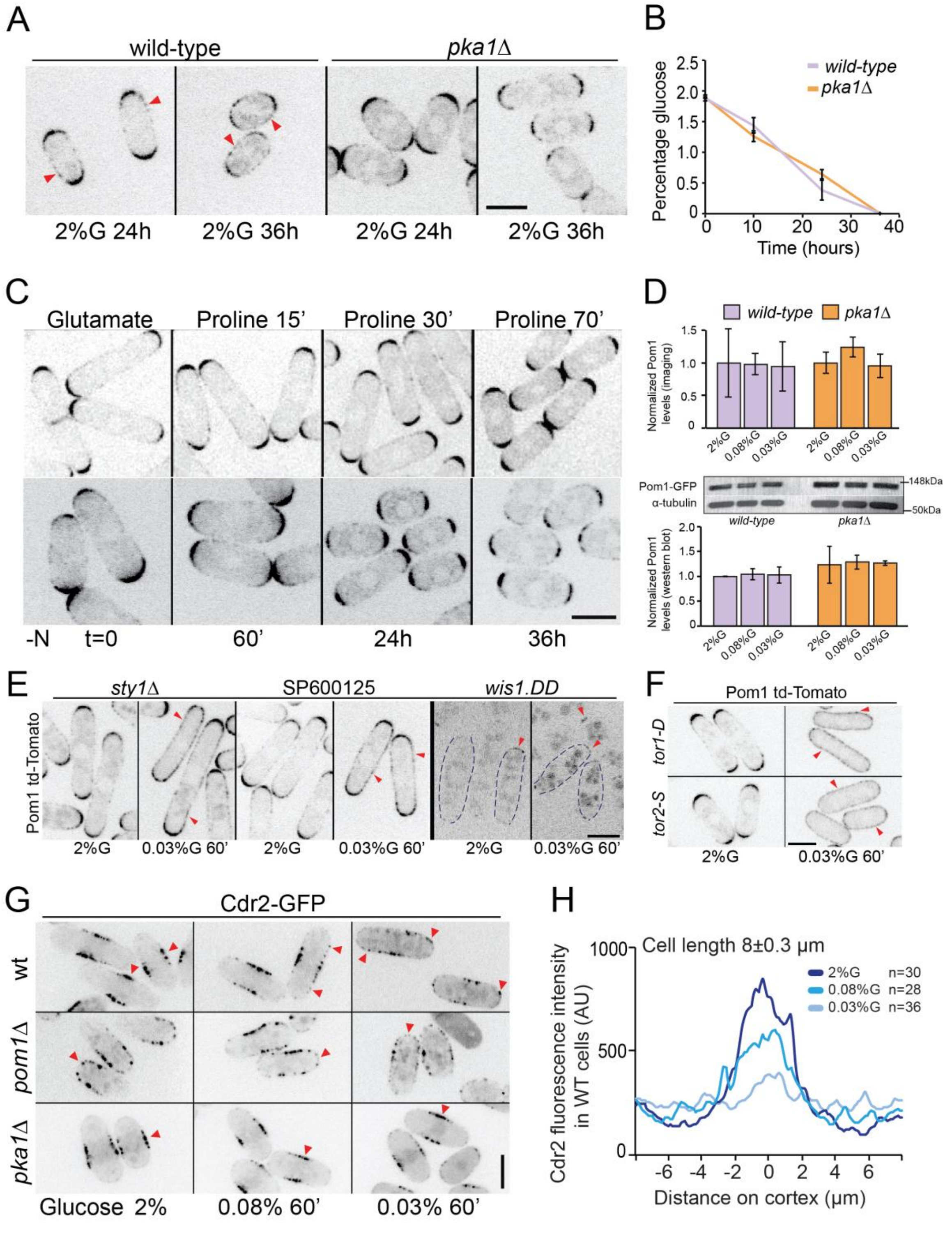

Supplementary Figure 1: Pom1 re-localization upon glucose limitation is specific to the PKA pathway

(A) Localization of Pom1-tdTomato in wild-type and *pka1*Δ cells grown to saturation. Arrowheads indicate Pom1 at cell sides in wild-type cells.

(B) Measurement of glucose levels in wild-type and *pka1*Δ cells grown to saturation. Average of 3 independent experiments is shown

(C) Localization of Pom1-tdTomato in wild-type cells before or after shift from glutamate to proline (poor nitrogen source) (top) or after nitrogen withdrawal (bottom) at indicated time points.

(D) Mean global Pom1-tdTomato levels in wild-type and *pka1*Δ cells grown in 2% or 0.08% or 0.03% glucose for 1h as measured by imaging (top, n>28) and Pom1-GFP levels as measured by western blotting. Levels were normalized to those in wild-type in 2% glucose. Average of 3 independent experiments is shown

(E) Localization of Pom1-tdTomato in *sty1*Δ*,* wild-type treated with 25mM SP600125 and *wis1*^{DD}. In cells lacking the fission yeast MAPK (*sty1*Δ*/spc1*Δ), or upon inhibition of Sty1 with the SP600125 MAPK inhibitor, Pom1 showed a mild re-localization phenotype upon shift to low glucose. Pom1 levels were drastically reduced in *wis1*^{DD} cells, in which Sty1 is constitutively activated.

(F) Localization of Pom1-tdTomato in *tor1-D and tor2-S* mutants in 2% or 0.03% glucose for 1h. Pom1 behaved normally in *tor2-S* and *tor1-D* mutant cells, described to show an altered response to cell size shortening in 0.08% glucose [1]. *tor2-S* and *tor1-D* mutants were first grown at 25°C and shifted to either 2% glucose or 0.03% glucose for 1h at 36°C. Images shown are medial spinning disk confocal sections. Arrowheads indicate Pom1 at cell sides.

(G) Sum of 5 medial spinning disk confocal images taken over 30 seconds of Cdr2-GFP in wild-type, *pom1*Δ and *pka1*Δ cells grown in 2% or 0.08% or 0.03% glucose (G) for 1h. Arrowheads indicate Cdr2 presence at cell cortex.

(H) Distribution of cortical Cdr2 from one cell tip to the other (0 = cell middle) in wild-type cells obtained with the Cellophane plugin. Average of (n= 30, 28 and 36 for 2%, 0.08% and 0.03% glucose) of profiles in 8µm-long cells. Profiles obtained from other cell lengths are similar. Representative images from 2 independent experiments with quantification of 1 is shown.

Scale bars represent 5µm. Error bars show standard deviation.

Figure S2

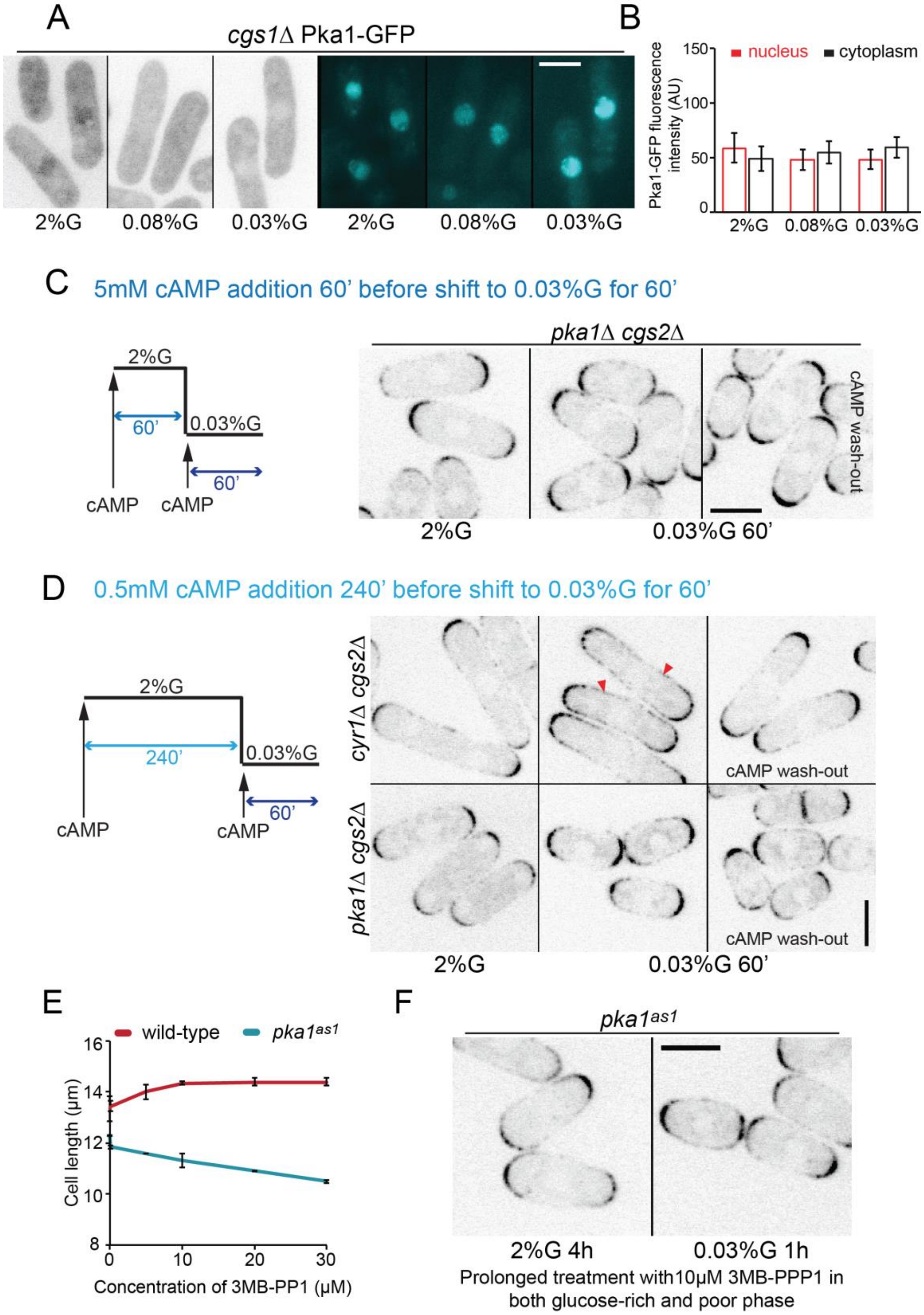

Supplementary Figure 2: Pka1 is active in low glucose to promote Pom1 side-localization

(A) Maximum intensity spinning disk projection of Pka1-GFP in *cgs1*Δ cells grown in 2% glucose or 0.08% or 0.03% glucose for 1h. Images show Pka1 in the GFP channel and Hoechst staining for chromatin in the UV channel (cyan).

(B) Measurement of cytoplasmic and nuclear Pka1-GFP levels in the same cells as in (A) (n>20). Experiments were performed thrice and quantification of one is shown

(C) Localization of Pom1-tdTomato in control *pka1*Δ *cgs2*Δ cells incubated with 5mM cAMP in 2% glucose (left panel) and shifted to 0.03% glucose with (middle) or without (right) cAMP.

(D) Localization of Pom1-tdTomato in *cyr1*Δ *cgs2*Δ and in control *pka1*Δ *cgs2*Δ cells incubated with 0.5mM cAMP in 2% glucose (left panel) and shifted to 0.03% glucose with (middle) or without (right) cAMP. Arrowheads indicate Pom1 at cell sides.

(E) Mean cell length at division of wild-type and *pka1-as1* cells treated with increasing 3MB-PP1 for 4h. (n>75). Error bars are standard deviations. Average of 3 independent experiments is shown.

(F) Localization of Pom1-tdTomato in *pka1-as1* cells treated with 10µM 3MB-PP1 in 2% glucose and imaged after 4h in 2% glucose or after 4h in 2% glucose + 1h in 0.03% G.

Representative medial spinning disk confocal images from 2-3 independent experiments are shown. Scale bars represent 5µm.

Figure S3

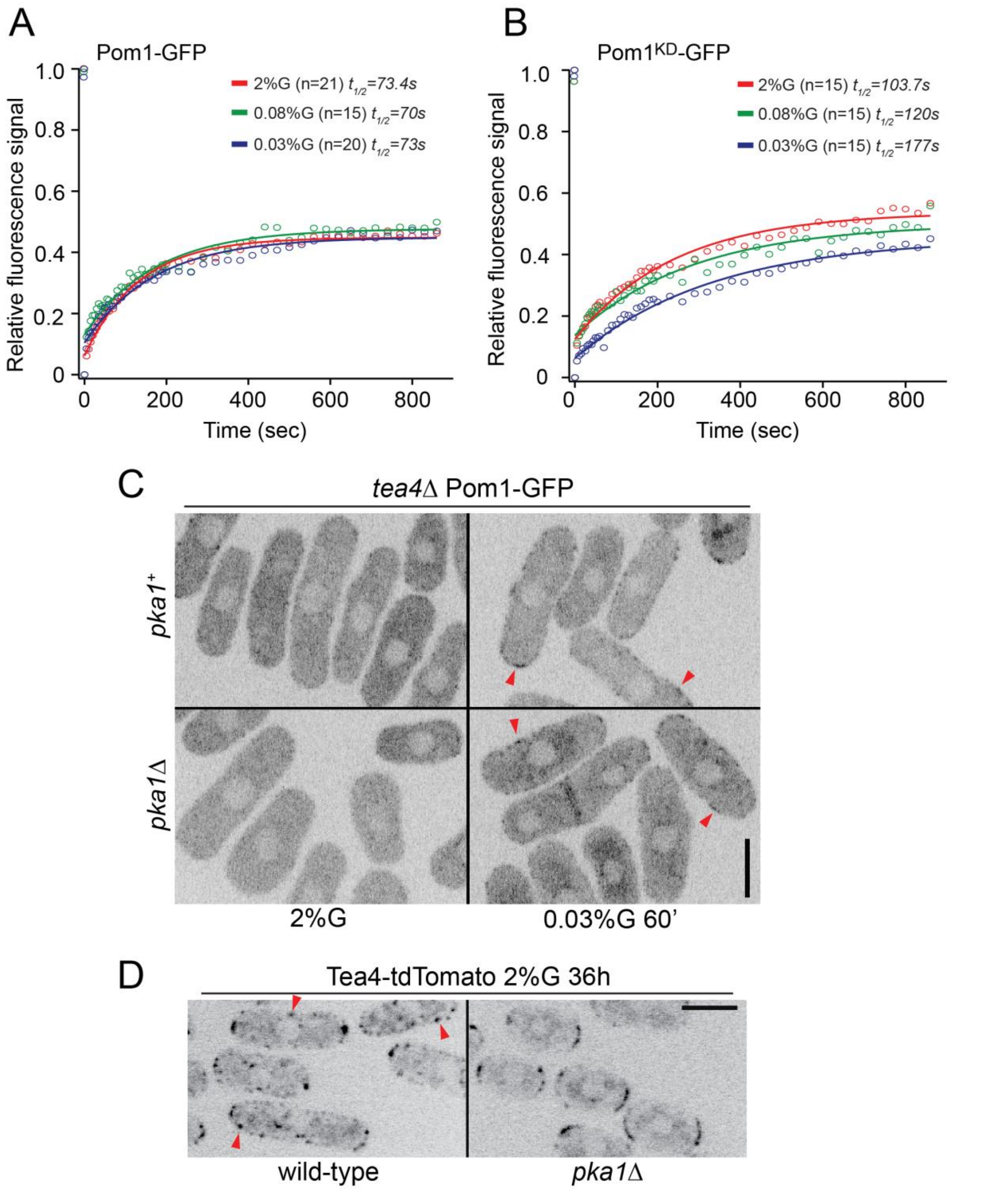

Supplementary Figure 3: Pom1 remains active and its localization depends on Tea4 upon glucose limitation

(A) Fluorescence recovery after photo-bleaching (FRAP) analysis on wild-type cells expressing Pom1-GFP grown in 2% glucose or 0.08% glucose or 0.03%

glucose for 1h. Previous work had shown that inactivation of Pom1 slows down its FRAP, yielding a longer recovery half-time, because inactive, un-phosphorylated Pom1 binds the plasma membrane more tightly than active Pom1 [2]. By contrast, glucose depletion did not alter Pom1 FRAP dynamics, which remained significantly higher than those of Pom1^{KD} (see panel B). This indicates Pom1 remains active in low glucose, and the side-localization observed is not due to a loss of Pom1's ability to auto-phosphorylate and detach from the membrane. (n=21, 15 and 20 tips)

(B) FRAP analysis on the Pom1^{KD}–GFP allele grown as in (A). This shows that Pom1^{KD} recovers significantly slower than wild-type Pom1 (see panel A). It also shows that the recovery half-time of Pom1^{KD} is significantly increased upon glucose limitation. Thus, glucose limitation increases the intrinsic affinity of Pom1 for the plasma membrane, independently of its phospho-regulation. This may be due to changes in the membrane potential. (n=15 tips)

Experiments were performed twice, quantification of one is shown

(C) Localization of Pom1-GFP in *tea4*Δ and *tea4*Δ *pka1*Δ cells grown in 2% glucose or 0.03% glucose for 1h. Previous work showed that Pom1 is cytosolic in *tea4*Δ cells in 2%G, because it cannot be dephosphorylated and thus fails to associate with the membrane. In 0.03%G, Pom1 also remained largely cytosolic, though a weak signal was detected at the cell cortex (arrowheads). We conclude that, in agreement with data shown in panel B, glucose limitation modestly increases the intrinsic affinity of Pom1 for the plasma membrane, possibly through changes in membrane potential. However, this modest effect is unlikely to explain the important redistribution observed in wild-type glucose-starved cells. In addition, the localization of Pom1-GFP was identical in *pka1*Δ *tea4*Δ, suggesting that the observed modest increase in Pom1 membrane-binding affinity in low-glucose is independent of Pka1 and that Pka1 requires Tea4 to modulate Pom1 localization.

(D) Localization of Tea4-tdTomato in wild-type and *pka1*Δ cells grown to saturation. Arrowheads indicate Tea4 dots present at cell sides.

Representative medial spinning disk confocal images from 2-3 independent experiments are shown. Scale bars represent 5µm.

Figure S4

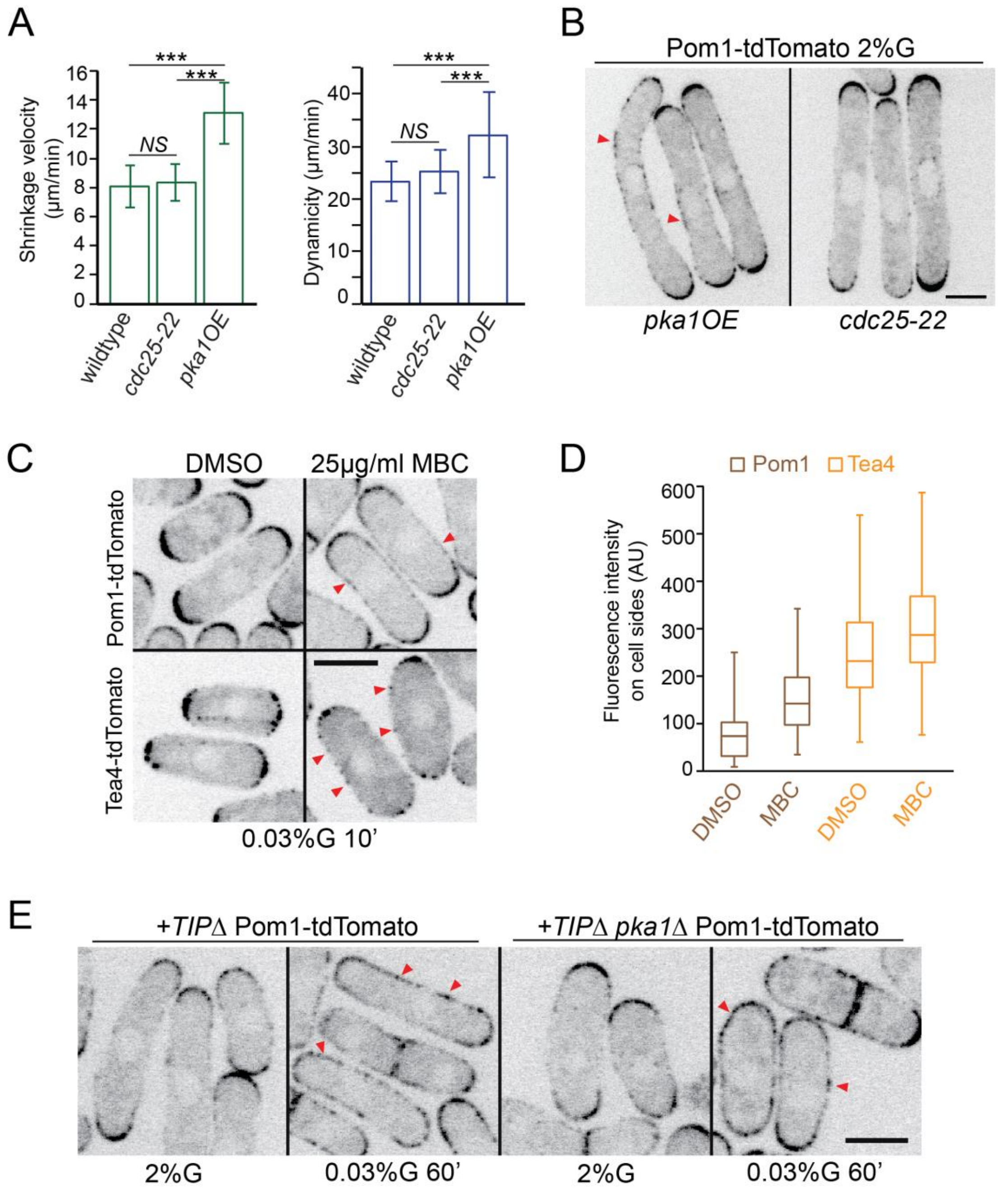

Supplementary Figure 4: Microtubule destabilization is sufficient to restore Tea4 and Pom1 side-localization in *pka1*Δ cells

(A) Mean microtubule shrinkage velocity (left) and dynamicity (right), in wild-type, *cdc25-22* and pka1OE cells grown in 2% glucose (n=30, n=20, n=24).

Statistical significance was derived using student's *t*-test. p=0.2, $p<10^{-11}$, $p<10^{-7}$). Error bars are standard deviations.

(B) Medial spinning disk confocal images of Pom1-tdTomato Pka1 over-expressing cells grown in 2% glucose (left). Elongated *cdc25-22* cells are used as control (right). Arrowheads mark Pom1 at cell sides.

(C) Sum of 5 medial spinning disk confocal images taken over 30 seconds of Pom1-tdTomato and Tea4-tdTomato in *pka1*Δ cells grown in 2%G, shifted to 0.03% glucose for 10min, and treated with DMSO (control) or 25µg/ml MBC at the time of shift. Arrowheads indicate Pom1 and Tea4 side localization in MBC treated cells.

(D) Box and whisker plot of cortical Pom1 and Tea4 fluorescence intensity in the middle 2µm region in *pka1*Δ cells treated as in (C). Experiment was repeated twice and quantification of one is shown.

(E) Medial spinning disk confocal images of Pom1-tdTomato in *mal3*Δ *tip1*Δ *tea2*Δ triple mutant (+*TIP*Δ) in *pka1*+ (left) or *pka1*Δ (right) cells grown in 2% glucose or shifted to 0.03% glucose for 1h. Arrowheads indicate Pom1 at cell sides. Similar results were obtained using *tip1*Δ single mutant in *pka1*+ or *pka1*Δ cells.

Representative images from 2 independent experiments are shown. Scale bars represent 5µm.

Figure S5

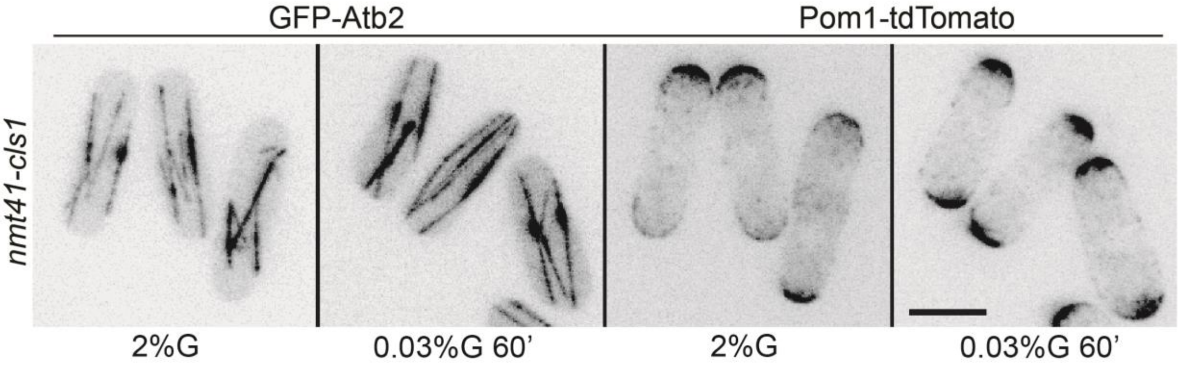

Supplementary Figure 5: Microtubule stabilization through CLASP over-expression mimics a *pka1*∆ phenotype

Maximum intensity spinning disk images of GFP-Atb2 and Pom1-tdTomato in cls1-overexpressing (*nmt41-cls1*) cells grown in 2% glucose or shifted to 0.03% glucose for 1h. *cls1* expression was induced by thiamine removal for 16-18h before low-glucose shift. Representative images from 2 independent experiments are shown. Scale bars represent 5µm.

Figure S6

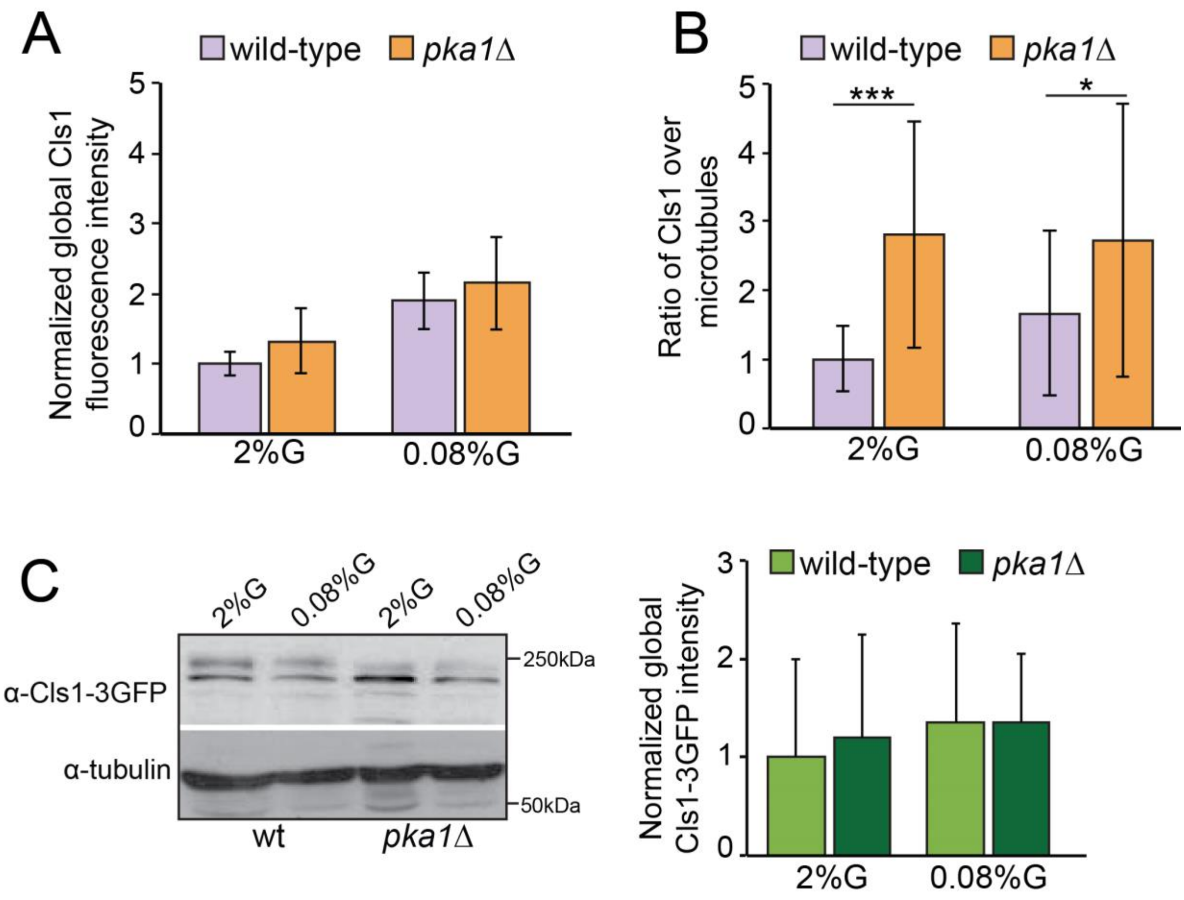

Supplementary Figure 6: Local and global Cls1-GFP levels in wild-type and *pka1*Δ cells.

(A) Mean global levels of Cls1-3GFP in wild-type and *pka1*Δ cells in 2% or 0.08% glucose, normalized to wild-type level in 2% glucose (n>26).

(B) Mean ratio between Cls1 and Atb2 local fluorescence on microtubule bundles in wild-type and *pka1*Δ cells in 2% or 0.08% glucose, normalized to wild-type level in 2% (n>27). Statistical significance was derived using student's *t*-test ($p<10^{-7}$, $p=0.016$).

(C) Western blot quantifications of Cls1-3GFP in wild-type and *pka1*Δ cells in 2% or 0.08% glucose, normalized to wild-type level in 2% glucose
Error bars are standard deviations. Average of 3 independent experiments is shown.

Supplementary Table 1: Strains used in this study

Strain number	Genotype	Source
Figure 1		
YSM1292	*h- pom1-tdTomato:natMX cdr2-mEGFP:kanMX ade6- leu1-32 ura4-D18*	[3]
YSM1554	*h+ pka1::ura4+ pom1-tdTomato-kanMX leu1-32 ura4-D18*	Lab stock
YSM1617	*pom1-tdTomato-natMX cdr2-GFP-kanMX git3::kanMX leu1-32*	Lab stock
YSM2365	*gpa2::ura4+ pom1-tdTomato-natMX ade6- leu1-32 ura4-*	This study
YSM 1553	*cyr1::LEU2+ pom1-tdTomato-kanMX leu1-32 ura4-D18*	Lab stock
YSM 1622	*pom1-tdTomato-natMX cgs1::ura4+ ade6- leu1-32 ura4-D18*	Lab stock
Figure 2		
YSM2417	*h+ pka1-GFP-kanMX ade6-M210 leu1-32 ura4-D18*	[4]
YSM2366	*h- cgs2::ura4+ cyr1::LEU2+ pom1-tdTomato-natMX leu1-32 ura4-*	This study
YSM2367	*$pka1^{as1}$ pom1-tdTomato-natMX leu1- ura4-*	This study
Figure 3		
YSM2368	*h- tea4-tdTomato-natMX ade6-M210 leu1-32 ura4-D18*	This study
YSM2369	*pka1::ura4+ tea4-tdTomato-natMX leu1- ura4-*	This study
Figure 4		
YSM2370	*h- leu1-32::pSV40-GFP-atb2:leu1+ ura4-*	Lab stock
YSM2371	*h+ pka1::ura4+ leu1-32::pSV40-GFP-atb2:leu1+ ura4-*	This study
YSM2372	*leu1-32::pSV40-GFP-atb2:leu1+ tea4-tdTomato-natMX ura4-*	This study
Figure 5		
YSM2373	*h+ cls1-36:ura4+ leu1-32::SV40-GFP-atb2:leu1+ ura4-*	This study
YSM2374	*pka1::ura4+ cls1-36:ura4+ leu1-32::SV40-GFP-atb2:leu1+ ura4-*	This study
YSM2370	*h- leu1-32::pSV40-GFP-atb2:leu1+ ura4-*	Lab stock

YSM2371	*h+ pka1::ura4+ leu1-32::pSV40-atb2-GFP:leu1+ ura4-*	This study
YSM2375	*cls1-36:ura4+ leu1-32::pSV40-atb2-GFP:leu1+ Tea4-tdTomato-natMX ura4-*	This study
YSM2376	*cls1-36:ura4+ pka1::ura4+ leu1-32::pSV40-atb2-GFP:leu1+ Tea4-tdTomato-natMX ura4-*	This study
YSM2377	*h+ cls1-36:ura4+ Pom1-tdTomato-natMX*	This study
YSM2378	*cls1-36:ura4+ pka1::hphMX Pom1-tdTomato-natMX*	This study
Figure 6		
YSM2379	*cls1-3GFP-kanMX mcherry-atb2 leu1-*	This study
YSM2380	*h- pka1::ura4+ cls1-3GFP-kanMX mcherry-atb2*	This study
YSM2381	*leu1-32::pSV40-GFP-atb2:leu1+ [pNnmt1-mcherry-cls1(1-500)]*	This study
YSM2382	*natMX-3nmt1-GFP-Pka1 leu1-32::pSV40-GFP-atb2:leu1+ [pNnmt1-mcherry-cls1(1-500)]*	This study
YSM2383	*natMX-3nmt1-GFP-Pka1 leu1-32::GFP-atb2:leu1+ ura4-*	This study
Supplementary Figure 1		
YSM1261	*h+ pom1-tdTomato-natMX ade6-M216 leu1-32 ura4-D18*	Lab stock
YSM1554	*h+ pka1::ura4+ pom1-tdTomato-kanMX leu1-32 ura4-D18*	Lab stock
YSM119	*h- pom1-GFP-kanMX ade6+ leu1+ura4+*	Lab stock
YSM2729	*pka1::ura4+ pom1-GFP-kanMX ade6+ leu1+*	This study
YSM1563	*sty1::ura4+ pom1-tdTomato-kanMX leu1-32 ura4-D18*	Lab stock
YSM1556	*wis1^{DD} pom1-td-Tomato-kanMX leu1-32 ura4-D18*	This study
YSM2384	*tor1D-hphMX pom1-tdTomato-natMX*	This study
YSM2385	*tor2S-kanMX pom1-tdTomato-natMX*	This study
YSM1292	*h- pom1-tdTomato:natMX cdr2-mEGFP:kanMX ade6- leu1-32 ura4-D18*	[3]
YSM2701	*pka1::ura4+ cdr2-GFP:kanMX*	This study
YSM1286	*h+ cdr2-GFP-kanMX pom1Δ::ura4+ ade6- leu1- ura4-*	Lab Stock
Supplementary Figure 2		

YSM2705	*cgs1Δ::ura4+ pka1-GFP-kanMX ade6- ura4-*	This study
YSM2386	*cgs2::ura4+ pka1::kanMX pom1-tdTomato-natMX ura4-*	This study
YSM2366	*h- cgs2::ura4+ cyr1::LEU2+ pom1-tdTomato-natMX leu1-32 ura4-*	This study
YSM1180	*h- ade6-M210 leu1-32 ura4-D18*	Lab stock
YSM2387	*h-* $pka1^{as1}$*(M278G) leu1-32 ura4-D18*	This study
YSM2367	$pka1^{as1}$ *pom1-tdTomato-natMX leu1- ura4-*	This study
Supplementary Figure 3		
YSM119	*h- pom1-GFP-kanMX ade6+ leu1+ ura4+*	[5]
YSM1511	*h-* $pom1^{KD}$*-GFP-kanMX ade6+ leu1+ ura4+*	[2]
YSM165	*h- tea4::kanMX pom1-GFP-kanMX ura4-*	[6]
YSM2388	*tea4::kanMX6 pom1-GFP-kanMX6 pka1::ura4+ ura4-*	This study
Supplementary Figure 4		
YSM2370	*h- leu1-32::pSV40-GFP-atb2:leu1+ ura4-*	Lab stock
YSM2389	*cdc25-22 aur-mcherry-atb2 leu1-*	This study
YSM2390	*h- kanMX-3nmt1-GFP-pka1 aur-mCherry-atb2 leu1- ura4-*	This study
YSM2391	*h+ kanMX-3nmt1-GFP-pka1 Pom1-tdTomato-natMX ade6-M216 leu1-32 ura4-D18*	This study
YSM2392	*cdc25-22 pom1-tdTomato-NatMX leu1-32*	This study
YSM1554	*h+ pka1::ura4+ pom1-tdTomato-kanMX leu1-32 ura4-D18*	Lab stock
YSM2369	*pka1::ura4+ tea4-tdTomato-natMX leu1- ura4-*	This study
YSM2393	*tea2::his3 mal3::his3 tip1::kanMX pom1-tdTomato-natMX ura4-*	This study
YSM2394	*tea2::his3 mal3::his3 tip1::kanMX pka1::ura4+ pom1-tdTomato-natMX ura4-*	This study
Supplementary Figure 5		
YSM2395	*h- kanMX: nmt41-cls1 leu1-32::SV40-GFP-atb2:leu1+ pom1-tdTomato-natMX ade6-M216 ura4-D18*	This study
YSM2396	*h- kanMX: nmt41-cls1 leu1-32::SV40-GFP-atb2:leu1+ pom1-tdTomato-natMX pka1::hphMX*	This study

	Supplementary Figure 6	
YSM2379	*cls1-3GFP-kanMX mcherry-atb2 leu1-*	This study
YSM2380	*h- pka1::ura4+ cls1-3GFP-kanMX mcherry-atb2*	This study
Table 1		
YSM2370	*h- leu1-32::pSV40-GFP-atb2:leu1+ leu1- ura4-*	Lab stock
YSM2371	*h+ pka1::ura4+ leu1-32::pSV40-GFP-atb2:leu1+ura4-*	This study
YSM2373	*h+ cls1-36:ura4+ leu1-32::SV40-GFP-atb2:leu1+ ura4-*	This study
YSM2374	*pka1::ura4+ cls1-36:ura4+ leu1-32::SV40-GFP-atb2:leu1+ ura4-*	This study
YSM2389	*cdc25-22 aur-mcherry-atb2 leu1-*	This study
YSM2390	*h- kanMX-3nmt1-GFP-Pka1 aur-mCherry-atb2 leu1- ura4-*	This study
YSM2397	*mal3::his3+ leu1-32::SV40-GFP-atb2:leu1+ ura4-*	This study
YSM2398	*mal3::his3+ pka1::ura4+ leu1-32::SV40-GFP-atb2:leu1+ ura4-*	This study
YSM2399	*tea2::his3+ leu1-32::SV40-GFP-atb2:leu1+ ura4-*	This study
YSM2400	*tea2::his3+ pka1::ura4+ leu1-32::SV40-GFP-atb2:leu1+ ura4-*	This study
YSM2402	*tip1::KanMX6+ leu1-32::SV40-GFP-atb2:leu1+ ura4*	This study
YSM2401	*tip1::KanMX6 pka1::ura4+ leu1-32::SV40-GFP-atb2:leu1+ ura4*	This study
YSM2415	*tea2::his3 mal3::his3 tip1::kanMX leu1-32::SV40-GFP-atb2:leu1+*	This study
YSM2416	*tea2::his3 mal3::his3 tip1::kanMX pka1::ura4+ leu1-32::SV40-GFP-atb2:leu1+*	This study
YSM2403	*alp14::kanMX leu1-32::SV40-GFP-atb2:leu1+*	This study
YSM2404	*alp14::kanMX pka1::ura4+ leu1-32::SV40-GFP-atb2:leu1+ ura4*	This study
YSM2405	*ase1::kanMX leu1-32::SV40-GFP-atb2:leu1+ ura4-*	This study
YSM2406	*h+ ase1::kanMX pka1::ura4+ leu1-32::SV40-GFP-atb2:leu1+ - ura4-*	This study
YSM2407	*dhc1::ura4+ leu1-32::SV40-GFP-atb2:leu1+ pom1-tdTomato-natMX*	This study
YSM2408	*dhc1::ura4+ pka1::hphMX leu1-32::SV40-GFP-atb2:leu1+ pom1-tdTomato-natMX*	This study

Table 2

YSM1372	*h- WT ade6+ leu1+ ura4+*	Lab stock
YSM2409	*h- pka1::kanMX ade6+ leu1+ ura4+*	This study
YSM2410	*h+ sty1::ura4+ ade6+ leu1+ ura4+*	This study
YSM2411	*h+ sty1::ura4+ pka1::kanMX ade6+ leu1+ ura4+*	This study
YSM2412	*h- pom1::kanMX ade6+ leu1+ ura4+*	This study
YSM2413	*pom1::kanMX pka1::kanMX ade6+ leu1+ ura4+*	This study
YSM2414	*pom1::KanMX6 sty1::ura4+ ade6+ leu1+ ura4+*	This study
YSM1476	*cdr2::ura4+ ade6+ leu1+ ura4+*	Lab stock
YSM1499	*pom1::kanMX cdr2::ura4+*	Lab stock
YSM2706	*pom1*^{6A} *ade6+ leu1+ ura4+*	Lab stock
YSM1495	*pom1*^{KD} *ade6+ leu1+ ura4+*	[3]
YSM2702	*tea4::kanMX ade6+ leu1+ ura4+*	This study
YSM2703	*tea4*$^{222\text{-}225RVXF\text{-}RAXA}$ *ade6+ leu1+ ura4+*	This study
YSM2226	*cdr2* $^{S755A\text{-}758A}$ *ade6+ leu1-32 ura4-D18*	[7]
YSM2234	*pom1Δ cdr2*$^{S755A\text{-}758A}$ *ade6+ leu1-32 ura4-D18*	[7]
YSM2224	h- *ade6+ leu1-32 ura4-D18*	[7]
YSM2229	*pom1Δ::kanMX6 ade6+ leu1-32 ura4-D18*	[7]
YSM2704	*ssp1::ura4+ ade6+ leu1+ ura4+*	This study
YSM2436	*h- cdr2*^{T166A} *ade6+ leu1+ ura4+*	[8]

Supplementary Table 2: Plasmids used in this study

Systematic name	Developed name	Details of construct expressed
pSM1590	*pSP72-(pka1ORF-^{M278G}-3'utr 618bp)*	Pka1ORF-^{M278G}+ 3'utr 618bp
pSM1043	*pFA6a-KanMX6-P3nmt1-GFP-pka1ORF(1-547) + pka1promoter (last 500bp)*	Pka1ORF+ Pka1 promoter
pSM1317	*pFA6a-NatMX6-P3nmt1-pka1ORF(1-547bp) + pka1promoter (last500bp)*	Pka1ORF+ Pka1 promoter
pSM1480	*pNmt1-mcherry-cls1(1-500)*	Cls1 (1-500)

Supplementary References

1. Ikai N, Nakazawa N, Hayashi T, Yanagida M. The reverse, but coordinated, roles of Tor2 (TORC1) and Tor1 (TORC2) kinases for growth, cell cycle and separase-mediated mitosis in Schizosaccharomyces pombe. *Open biology* **1**, 110007 (2011).

2. Hachet O, Berthelot-Grosjean M, Kokkoris K, Vincenzetti V, Moosbrugger J, Martin SG. A phosphorylation cycle shapes gradients of the DYRK family kinase Pom1 at the plasma membrane. *Cell* **145**, 1116-1128 (2011).

3. Martin SG, Berthelot-Grosjean M. Polar gradients of the DYRK-family kinase Pom1 couple cell length with the cell cycle. *Nature* **459**, 852-856 (2009).

4. Matsuo Y, McInnis B, Marcus S. Regulation of the subcellular localization of cyclic AMP-dependent protein kinase in response to physiological stresses and sexual differentiation in the fission yeast Schizosaccharomyces pombe. *Eukaryot Cell* **7**, 1450-1459 (2008).

5. Bähler J, Pringle JR. Pom1p, a fission yeast protein kinase that provides positional information for both polarized growth and cytokinesis. *Genes Dev* **12**, 1356-1370 (1998).

6. Padte NN, Martin SG, Howard M, Chang F. The cell-end factor pom1p inhibits mid1p in specification of the cell division plane in fission yeast. *Curr Biol* **16**, 2480-2487 (2006).

7. Bhatia P*, et al.* Distinct levels in Pom1 gradients limit Cdr2 activity and localization to time and position division. *Cell Cycle* **13**, (2013).

8. Deng L, Baldissard S, Kettenbach AN, Gerber SA, Moseley JB. Dueling kinases regulate cell size at division through the SAD kinase Cdr2. *Curr Biol* **24**, 428-433 (2014).
